# Antibodies targeting epitopes on the cell-surface form of NS1 protect against Zika virus infection during pregnancy

**DOI:** 10.1038/s41467-020-19096-y

**Published:** 2020-10-19

**Authors:** Alex W. Wessel, Nurgun Kose, Robin G. Bombardi, Vicky Roy, Warangkana Chantima, Juthathip Mongkolsapaya, Melissa A. Edeling, Christopher A. Nelson, Irene Bosch, Galit Alter, Gavin R. Screaton, David H. Fremont, James E. Crowe, Michael S. Diamond

**Affiliations:** 1 Department of Pathology and Immunology, Washington University School of Medicine, St. Louis, MO 63110 USA; 2 Departments of Pediatrics, Pathology, Microbiology and Immunology, Vanderbilt University Medical Center, Nashville, TN 37232 USA; 3 Vanderbilt Vaccine Center, Vanderbilt University Medical Center, Nashville, TN 37232 USA; 4 Ragon Institute of MGH, MIT, and Harvard University, Cambridge, MA 02139 USA; 5 Nuffield Department of Medicine, Wellcome Centre for Human Genetics, University of Oxford, Oxford, OX3 7BN UK; 6 Dengue Hemorrhagic Fever Unit, Faculty of Medicine, Office for Research and Development, Siriraj Hospital, Mahidol University, Bangkok, 10700 Thailand; 7 E25Bio, Inc., The Engine of MIT, Cambridge, MA 02139 USA; 8 Department of Molecular Microbiology, Washington University School of Medicine, St. Louis, MO 63110 USA; 9 Department of Biochemistry and Molecular Biophysics, Washington University School of Medicine, St. Louis, MO 63110 USA; 10 The Andrew M. and Jane M. Bursky Center for Human Immunology and Immunotherapy Programs, Washington University School of Medicine, St. Louis, MO 63110 USA; 11 Department of Medicine, Washington University School of Medicine, St. Louis, MO 63110 USA

**Keywords:** Viral infection, Dengue virus, Virus-host interactions

## Abstract

There are no licensed therapeutics or vaccines available against Zika virus (ZIKV) to counteract its potential for congenital disease. Antibody-based countermeasures targeting the ZIKV envelope protein have been hampered by concerns for cross-reactive responses that induce antibody-dependent enhancement (ADE) of heterologous flavivirus infection. Nonstructural protein 1 (NS1) is a membrane-associated and secreted glycoprotein that functions in flavivirus replication and immune evasion but is absent from the virion. Although some studies suggest that antibodies against ZIKV NS1 are protective, their activity during congenital infection is unknown. Here we develop mouse and human anti-NS1 monoclonal antibodies that protect against ZIKV in both non-pregnant and pregnant mice. Avidity of antibody binding to cell-surface NS1 along with Fc effector functions engagement correlate with protection in vivo. Protective mAbs map to exposed epitopes in the wing domain and loop face of the β-platform. Anti-NS1 antibodies provide an alternative strategy for protection against congenital ZIKV infection without causing ADE.

## Abstract (web summary)

Zika virus is an arthropod-transmitted flavivirus that can cause microcephaly and other fetal abnormalities during pregnancy. Here Wessel et al. develop antibodies against the Zika virus nonstructural protein 1 that protect non-pregnant and pregnant mice against infection, and define particular antibody epitopes and mechanisms underlying this protection.

## Introduction

Zika virus (ZIKV) is an arthropod-transmitted flavivirus that historically caused sporadic human infections in Africa and Asia after its discovery in 1947^1^. However, its recent dissemination to Oceania and the Americas drew global attention due to its association with new and severe clinical manifestations^2^. Whereas most ZIKV infections are asymptomatic or present as a mild febrile illness, the epidemic in French Polynesia established a linkage to severe neurological complications including Guillain-Barré syndrome^3–5^. In Brazil and other countries of the Americas, infection during pregnancy caused microcephaly and other congenital malformations^6,7^. Although the epidemic has waned, the potential for re-emergence of ZIKV poses a significant threat to public health. Nonetheless, there are no approved vaccine or therapeutic countermeasures.

ZIKV is related closely to other pathogenic flaviviruses, including the four serotypes of dengue (DENV), West Nile (WNV), Japanese encephalitis (JEV), yellow fever (YFV), and tick-borne encephalitis (TBEV) viruses. Flavivirus NS1 is a highly conserved 48 kDa glycoprotein that dimerizes upon translocation into the endoplasmic reticulum, where it fulfills a scaffolding function in viral RNA replication^8–10^. NS1 also is expressed on the plasma membrane of infected cells as a dimer^11,12^ and is secreted into the extracellular space as a soluble hexamer^13^. The cell surface and soluble forms of NS1 modulate host immunity through interactions with complement proteins^14–17^ and possibly Toll-like receptors (TLRs)^18,19^. Soluble NS1 accumulates in the serum of flavivirus-infected human subjects^20–22^, which reportedly enhances infectivity of virus transmitted to mosquito vectors during a blood meal^23,24^. Soluble NS1 also can bind back to the surface of uninfected or infected cells, and this activity may impact endothelial integrity and permeability at blood-tissue barriers^25–28^. The significance of these findings to pathogenesis, however, remains uncertain^13^.

NS1 is comprised of three distinct domains: an N-terminal β-roll domain (residues 1–29), a wing domain (residues 30–180), and a β-platform domain (residues 181–352), which has two faces, one of β-strands and a second largely composed of an extended loop, termed the spaghetti loop (residues 219–272)^29,30^. Following translation in the ER, NS1 dimerizes via intertwining of the β-roll domains from two protomers. The dimer creates a surface for membrane interaction via conserved hydrophobic residues within the β-roll domain and flexible loop (residues 108–129) and “greasy finger” (residues 159–163) regions of the wing domain^29,31,32^. This hydrophobic surface also facilitates trimerization of dimers into the NS1 hexamer, which contains an inner hydrophobic channel that is rich in lipids^33^. Other regions of the wing and β-platform domains contribute to forming the electrostatic exterior surface of the hexamer and the membrane-distal surface of the dimer.

Monoclonal antibodies (mAbs) against NS1 can confer protection against WNV, JEV, and YFV in animal models^34–36^. Passive transfer of a single NS1-specific human mAb or polyclonal antibodies elicited by an NS1 DNA vaccine protected against lethal ZIKV challenge in *Stat2*^*−/−*^ mice^37,38^. Although anti-NS1 mAbs have been developed against multiple flaviviruses, few studies have mapped their epitopes or defined the mechanisms of action. Here we generate murine and human mAbs against ZIKV NS1 and assess their efficacy in vivo in immunocompetent human STAT2 knock-in (hSTAT2 KI) and immunocompromised wild-type mice^39^. Four murine mAbs (Z11, Z15, Z17, and Z18) and three human mAbs (749-A4, ZIKV-231, and ZIKV-292) confer protection against ZIKV in non-pregnant mice by limiting viral infection. A subset of these mAbs also confer protection to the developing fetus following virus inoculation of pregnant mice. Protection in vivo by anti-NS1 mAbs correlates with strength and density of binding to NS1 on the cell surface and depends on Fc interactions, as a variant antibody that cannot engage Fc-γ receptors or complement loses protective activity. Mapping analyses reveals that the protective mAbs bind to one of two epitopes: (i) the exposed hydrophilic surface of the wing domain or (ii) the C-terminal tip of the loop face of the β-platform domain. Our findings suggest that antibodies recognizing epitopes that are accessible on cell surface forms of NS1 confer protection against ZIKV in vivo, including during pregnancy.

## Results

### Generation of anti-ZIKV NS1 mAbs

To generate murine mAbs against NS1, we inoculated and boosted BALB/c mice with a mouse-adapted variant of ZIKV Dakar 41525 (Senegal, 1984)^39^. To focus the humoral response on NS1, we also boosted mice two additional times with adjuvanted recombinant full-length ZIKV NS1 protein. We isolated 42 hybridomas that secreted NS1-binding mAbs as determined by enzyme-linked immunosorbent assay (ELISA) with recombinant NS1 protein and flow cytometry of ZIKV-infected cells (Supplementary Table 1). Ten antibodies of the IgG2a subclass were prioritized given prior evidence of the protective activity of anti-WNV NS1 mAbs of this isotype^35^. One IgG2a mAb, Z21, displayed weak binding to ZIKV NS1 and was not studied further.

We separately generated human mAbs against ZIKV NS1 from B cells of subjects who had been infected with ZIKV^40^. We isolated 22 human mAbs that bound to ZIKV NS1 protein by ELISA. Initially, we tested the human mAbs in a stringent, lethal challenge model of ZIKV in immunocompromised mice^41^. Two of the 22 human mAbs, ZIKV-231 and ZIKV-292, conferred some protection against lethality (12% and 40% survival, respectively, compared to 0% of controls) and were prioritized for further testing. One additional human IgG1, 749-A4, was isolated from a DENV-immune subject and was prioritized, because it cross-reacted with ZIKV NS1.

We evaluated 10 murine IgG2a and all 23 human IgG mAbs for more extensive cross-reactivity with NS1 proteins of ZIKV, DENV serotype 2 (DENV2), WNV, JEV, TBEV, and YFV (Supplementary Table 2). All of the murine IgG2a and 20 of the 23 human IgG mAbs were ZIKV-specific, whereas human mAbs ZIKV-240 and ZIKV-315 demonstrated weak reactivity with JEV and DENV2, respectively. However, human mAb 749-A4 bound to ZIKV, DENV2, WNV, and JEV NS1, but not to TBEV or YFV NS1. In comparison, 9NS1, a previously generated mouse IgG1 mAb against WNV NS1^35^, recognized all six flavivirus NS1 proteins tested.

We performed an ELISA-based binding assay to define competition groups for the 10 murine IgG2a and 3 prioritized human IgG1 mAbs (Supplementary Tables 2 and 3). The mAbs segregated into five competition groups: group A, Z12, Z13, and Z19; group B, Z11, Z15, Z18, and ZIKV-292; group C, Z17, ZIKV-231, and 749-A4; group D, Z14 and Z16; and group E, Z20.

### Protection by anti-ZIKV NS1 mAbs in immunocompetent and immunocompromised mice

**Fig. 1:**
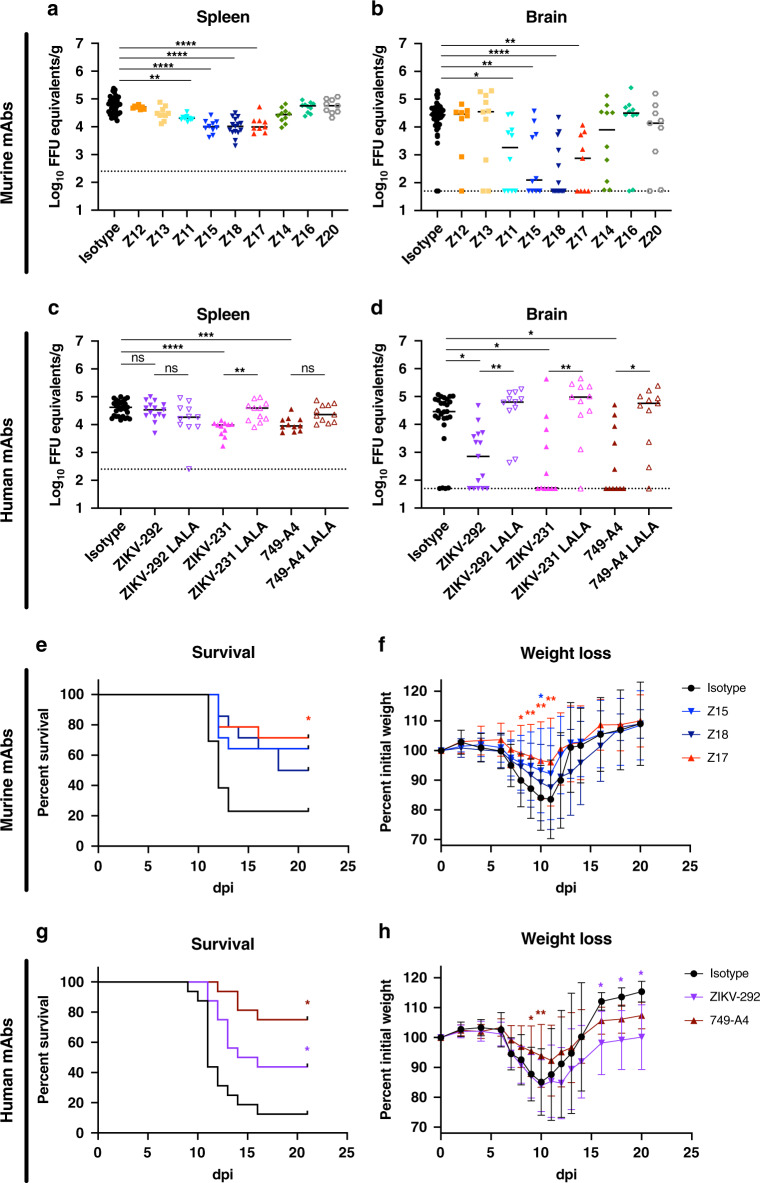
Protection by anti-ZIKV NS1 mAbs in non-pregnant mice. Three- to 4-week-old male and female hSTAT2 KI mice were administered 200 μg of the indicated murine (**a**, **b**) or human (**c**, **d**) mAbs and inoculated subcutaneously with 10^5^ FFU of ZIKV. Viral burden was assessed at 9 dpi in the spleen (**a**, **c**) and brain (**b**, **d**). **a**, **b** Isotype control, *n* = 33; Z12, *n* = 8; Z13, *n* = 10; Z11, *n* = 10; Z15, *n* = 10; Z17, *n* = 9; Z18, *n* = 15; Z14, *n* = 10; Z16, *n* = 10; Z20, *n* = 9. **c**, **d** Isotype control, *n* = 27; 749-A4, *n* = 11; 749-A4 LALA, *n* = 11; ZIKV-231, *n* = 11; ZIKV-231 LALA, *n* = 11; ZIKV-292, *n* = 15; ZIKV-292 LALA, *n* = 11. Data are pooled from at least two experiments (Kruskal–Wallis one-way ANOVA with Dunn’s post-test comparison between the indicated groups; *p < 0.05; **p < 0.01; ***p < 0.001; ****p < 0.0001; **a** Z11, *p* = 0.002; **b** Z11, *p* = 0.015; Z15, *p* = 0.006; Z17, *p* = 0.003; **c** isotype vs. 749-A4, *p* = 0.0001; ZIKV-231 vs. ZIKV-231 LALA, *p* = 0.004; **d** isotype vs. ZIKV-231, *p* = 0.039; isotype vs. ZIKV-292, *p* = 0.012; isotype vs. 749-A4, *p* = 0.013; ZIKV-231 vs. ZIKV-231 LALA, *p* = 0.004; ZIKV-292 vs. ZIKV-292 LALA, *p* = 0.003; 749-A4 vs. 749-A4 LALA, *p* = 0.013). Dotted lines denote the limit of detection (LOD) of the assay. **e**–**h** Four- to 5-week-old male C57BL/6J mice administered 1 mg of an anti-Ifnar1-blocking mAb and 500 μg of the indicated murine (**e**, **f**) or human (**g**, **h**) mAbs. The following day, mice were inoculated subcutaneously with ZIKV and mortality (**e**, **g**) and weights (**f**, **h**) were tracked. Data are representative of two experiments and survival analysis was performed using the Mantel–Cox log-rank test with Bonferroni correction compared to the isotype group (**e** isotype, *n* = 13; Z15, *n* = 14, *p* = 0.052; Z17, *n* = 14, *p* = 0.018; Z18, *n* = 14, *p* = 0.089; **g**: isotype, *n* = 16; ZIKV-292, *n* = 16, *p* = 0.026; 749-A4, *n* = 16, *p* < 0.002). In the figure: *p < 0.05, **p < 0.01. Weights were analyzed by two-way ANOVA with Dunnett’s post-test comparison to isotype-treated animals (*p < 0.05; **f** Z15: day 10, *p* = 0.037; Z17: day 8, *p* = 0.037; day 9, *p* = 0.009; day 10, *p* = 0.002; day 11, *p* = 0.006; **h** 749-A4: day 9, *p* = 0.014; day 10, *p* = 0.005; ZIKV-292: day 16, *p* = 0.045; day 18, *p* = 0.037; day 20, *p* = 0.027); error bars represent the mean values ± SD. Source data are provided as a Source Data file for Figs. 1f, h.

We tested the efficacy of anti-NS1 mAbs against ZIKV in immunocompetent, male and female hSTAT2 KI mice. These animals support infection, because ZIKV can antagonize human STAT2 but not mouse Stat2^39^. The hSTAT2 KI mouse model is more typical of moderate human infection and does not support the severe ZIKV disease seen with immunocompromised mice: 3-week-old infected mice have limited lethality and fetuses from infected pregnant mice sustain high levels of ZIKV but do not develop microcephaly or fetal demise^7,39,41^. Three- to 4-week-old mice received a single 200 μg dose (~10 mg/kg) of anti-ZIKV NS1 or isotype control mAb via intraperitoneal injection concurrently with subcutaneous inoculation of ZIKV in the foot. Whereas murine mAbs Z12, Z13, Z14, Z16, and Z20 failed to diminish viral burden in the spleen or brain at 9 days post-infection (dpi), Z11, Z15, Z18, and Z17 reduced ZIKV infection in both tissues, with several animals having viral RNA levels below the limit of detection (Fig. 1a, b). Human mAbs 749-A4 and ZIKV-231 conferred similar reductions in viral burden in the spleen and brain, whereas ZIKV-292 protected only in the brain (Fig. 1c, d). No mAb treatment completely protected, as viral RNA breakthrough was observed in a subset of animals.

Previous studies with some anti-WNV and anti-YFV NS1 mAbs showed that Fc effector functions were required for optimal protection^34,35^. To test for effects on protection against ZIKV, we engineered leucine (L) to alanine (A) substitutions at residues 234 and 235 (LALA) for three of the human mAbs (ZIKV-231, ZIKV-292, and 749-A4) and confirmed diminished binding to FcγRs by direct ELISA (Supplementary Fig. 1a). When hSTAT2 KI mice were administered the anti-NS1 mAb IgG-LALA variants and inoculated with ZIKV, we observed no protection in the brain compared to the isotype control mAb-treated mice, consistent with a contribution of Fc effector functions (Fig. 1d).

For a subset of the mAbs that protected in hSTAT2 KI mice, we assessed their ability to prevent lethal ZIKV infection using a more stringent immunocompromised mouse model^39,41^. Four- to 5-week-old C57BL/6J mice were administered 1 mg of anti-Ifnar1-blocking mAb and 500 μg (~25 mg/kg) of anti-ZIKV NS1 or isotype control mAb via intraperitoneal injection. The following day, mice were inoculated with ZIKV via subcutaneous injection in the foot, and lethality and weights were recorded for 21 days. Of the three murine IgG2a mAbs tested, Z17 significantly protected against ZIKV-mediated lethality and weight loss (Fig. 1e, f). Although Z15 and Z18 conferred partial protection against lethality, these results did not attain statistical significance (Z15, *p* = 0.052; Z18, *p* = 0.089). Of the two human IgG1 mAbs tested in this model, both 749-A4 and ZIKV-292 prevented lethality, but only 749-A4 limited weight loss (Fig. 1g, h).

### Antibody protection in pregnant mice

**Fig. 2:**
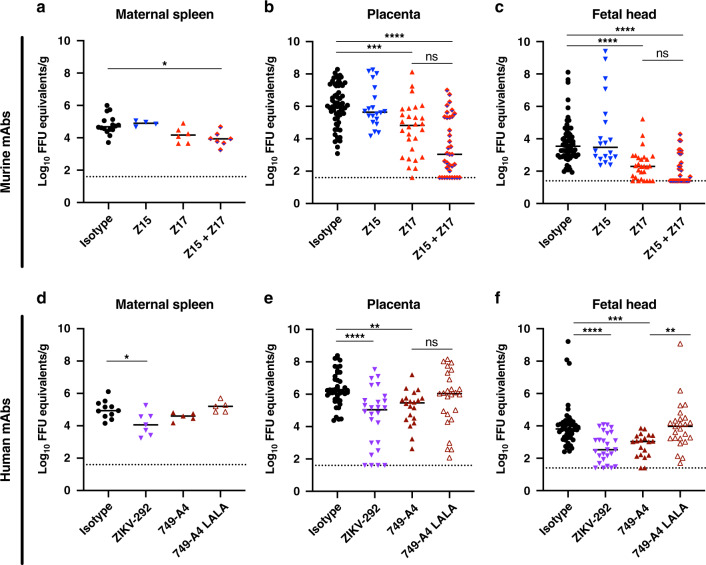
Protection by anti-ZIKV NS1 mAbs in pregnant hSTAT2 KI mice. Eight- to 16-week-old hSTAT2 KI female and male mice were mated. On E6.5, plugged females were administered 250 μg of the indicated murine (**a**–**c**) or human (**d**–**f**) mAbs and inoculated subcutaneously with 10^6^ FFU of ZIKV. For experiments with antibody cocktail treatment, mice received 125 μg of each mAb. Viral burden was assessed on E13.5 in maternal spleen (**a**, **d**), placenta (**b**, **e**), and fetal head (**c**, **f**). Viral burden data for the maternal spleen includes mice that were plugged but did not become pregnant. For each pregnant dam, five placentas and fetal heads were evaluated unless the total number of fetuses was less than 5. **a** Isotype, *n* = 16; Z15, *n* = 4; Z17, *n* = 6; Z15 + Z17, *n* = 7. **b**, **c** Isotype, *n* = 61; Z15 *n* = 20; Z17, *n* = 28; Z15 + Z17, *n* = 35. **d** Isotype, *n* = 9; ZIKV-292, *n* = 4; 749-A4 and 749-A4 LALA, *n* = 5. **e**, **f** Isotype, *n* = 45; ZIKV-292, *n* = 26; 749-A4, *n* = 20; 749-A4 LALA, *n* = 25. Data are pooled from at least three experiments (Kruskal–Wallis one-way ANOVA with Dunn’s post-test comparison between the indicated groups; *p < 0.05; **p < 0.01; ***p < 0.001; ****p < 0.0001; **a** isotype vs. Z15 + Z17, *p* = 0.010; **b** isotype vs. Z17, *p* = 0.0006; isotype vs. Z15 + Z17, *p* < 0.0001; **c** isotype vs. Z17, *p* < 0.0001; isotype vs. Z15 + Z17, *p* < 0.0001; **d** isotype vs. ZIKV-292, *p* = 0.048; **e** isotype vs. ZIKV-292, *p* < 0.0001; isotype vs. 749-A4, *p* = 0.004; **f** isotyp**e** vs. ZIKV-292, *p* < 0.0001; isotype vs. 749-A4, *p* = 0.0003; 749-A4 vs. 749-A4 LALA, *p* = 0.002). Dotted lines denote the LOD.

We tested whether anti-NS1 mAbs could protect against ZIKV during pregnancy. We used hSTAT2 KI mice, in which ZIKV infection during pregnancy results in transmission to the fetal brain without fetal demise^39^. For the murine mAbs, we focused efforts on testing Z15 (group B) and Z17 (group C), given their protection in young hSTAT2 KI mice and distinct competition groups (Fig. 2a–c). We tested two human mAbs: ZIKV-292 from competition group B and 749-A4 from group C (Fig. 2d–f). After mating adult male and female hSTAT2 KI mice, at embryo day (E)6.5, females were inoculated subcutaneously with ZIKV and concurrently administered 250 μg of anti-NS1 or isotype control mAb via intraperitoneal injection. Pregnant dams were euthanized on E13.5, and viral RNA levels were measured in the maternal spleen, the fetal-derived placenta, and fetal head. Despite conferring protection in non-pregnant, young hSTAT2 KI mice, murine mAb Z15 did not reduce viral burden in maternal or fetal tissues (Fig. 2a–c). Administration of mAb Z17, however, markedly reduced ZIKV infection in the placenta and fetal head (Fig. 2b, c). To determine whether a combination of mAbs from different competition groups could enhance protection during pregnancy, we administered pregnant mice a cocktail of Z15 (125 μg) and Z17 (125 μg) mAbs. Treatment with the mAb cocktail trended toward greater reduction in ZIKV titers in the placenta and fetal head compared to treatment with Z17 alone (51% vs. 21% of samples at the limit of detection in the fetal head), although this difference did not attain statistical significance.

Treatment of pregnant dams with human mAb ZIKV-292 (group B) also reduced viral burden in the placenta and fetal head (Fig. 2d–f). Group C mAb 749-A4 conferred a small yet statistically significant reduction of virus in these tissues as well. Notably, the protective effect of 749-A4 was lost when an IgG-LALA variant was used, which suggests a role for Fc effector functions in anti-NS1 antibody-mediated protection during pregnancy.

### Avidity for cell surface NS1 correlates with protection

**Fig. 3:**
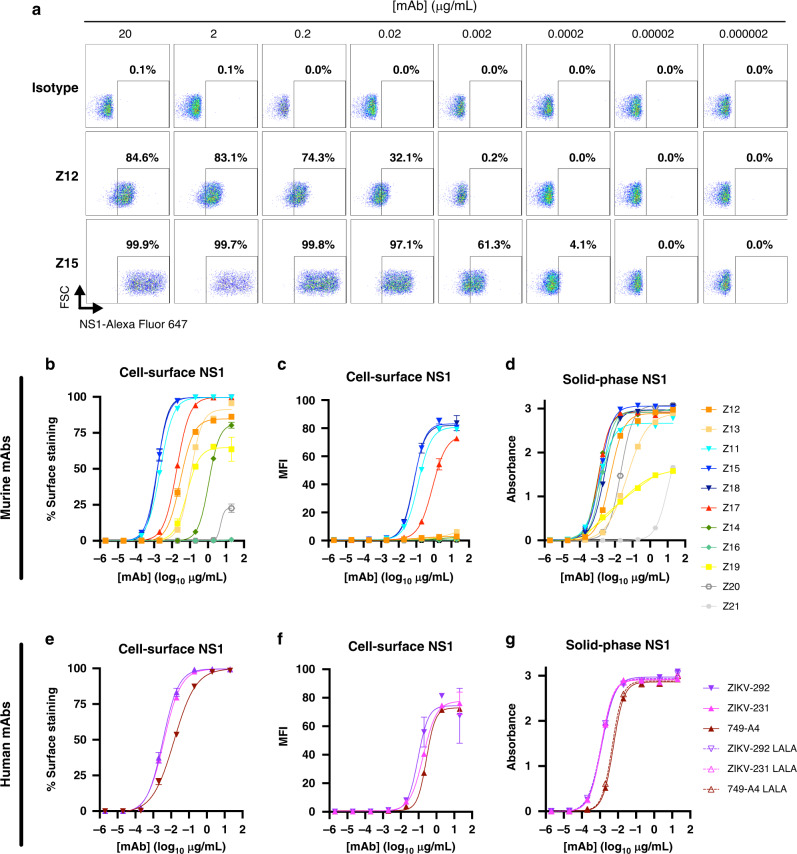
Binding properties of anti-ZIKV NS1 mAbs. Binding to NS1 by anti-NS1 mAbs. Binding to cell surface-associated NS1 was assessed by flow cytometry following staining at 4 °C of live, ZIKV-infected Vero cells with serial dilutions of murine (**a**–**c**) or human (**e**, **f**) mAbs. **a** Representative flow cytometry plots showing binding of anti-NS1 mAbs to NS1 on the cell surface. A minimum of 5000 cells was collected per sample. The percentage of cells staining (**b**, **e**) or the mean fluorescence intensity (MFI) (**c**, **f**) is shown as the average of two replicates and is representative of two or three experiments; error bars represent the mean ± SD. **d**, **g** Binding of mAbs to recombinant, soluble NS1 was assessed by ELISA. Absorbance values for each concentration of mAb are the average of duplicates and representative of two experiments. EC_50_ values for binding are reported in Supplementary Table S2. Source data are provided as a Source Data file for Fig. 3b–g.

Given the Fc effector function-dependence of protection of some of our mAbs, we hypothesized that protective anti-ZIKV NS1 mAbs would recognize cell surface forms of NS1 to enable antibody-dependent immune cell clearance of virus-infected cells. We evaluated this idea using a flow cytometric assay with intact, ZIKV-infected cells. Murine mAbs Z11, Z15, and Z18, which protected against ZIKV in non-pregnant hSTAT2 KI mice, bound avidly to the surface of infected cells (half maximal effective concentration (EC_50_): Z11, 2.2 ng/mL; Z15, 1.5 ng/mL; Z18, 1.5 ng/mL; Fig. 3a, b and Supplementary Table 2). Murine mAb Z17, which also protected in vivo, bound slightly less avidly than Z15 and Z18 (EC_50_ of 18.4 ng/mL) but greater than non-protective murine mAbs Z12, Z13, and Z19 (EC_50_: Z12, 29.2 ng/mL; Z13, 105.7 ng/mL; Z19, 66.2 ng/mL). As Fc effector function-dependent protection likely requires antibody cross-linking to low-affinity Fc-γ receptors, which depends on both the strength of mAb binding to target antigens and the number of sites bound^42–46^, we examined the density of mAb binding to the cell surface using the mean fluorescence intensity. Antibody-binding density was substantially lower for the non-protective mAbs compared to the protective mAbs Z11, Z15, Z18, and Z17 (Fig. 3c). The non-protective murine mAbs had reduced avidity and density of binding (Z14), or did not show binding to NS1 on the surface of infected cells (Z16 and Z20). The human mAbs ZIKV-231, ZIKV-292, and 749-A4 bound to NS1 on the cell surface NS1 to a similar extent as mAb Z17 (EC_50_ of 3.9, 3.4, and 14.6 ng/mL, respectively; Fig. 3e, f). Despite their differential binding patterns to NS1 on the cell surface, all murine and human mAbs, except for Z19 and Z21, bound strongly to solid-phase, hexameric ZIKV NS1 in an ELISA (Fig. 3d, g).

### Effector functions of anti-NS1 mAbs

To begin to understand potential Fc effector mechanisms of protection, we assessed the activity of a subset of our anti-ZIKV NS1 mAbs using in vitro effector function assays. To assess antibody-dependent complement deposition, we coupled beads to recombinant ZIKV NS1, added anti-NS1 mAbs, and measured complement (C3b) deposition. Among the anti-NS1 mAbs tested, Z17, ZIKV-292, and 749-A4 promoted complement (C3b) deposition (Supplementary Fig. 1b). All of the mAbs tested also promoted internalization of NS1 antigen-coated beads by neutrophils or monocytes (Supplementary Fig. 1c, d).

### Protective mAbs bind to distinct epitopes in NS1

To begin to understand the basis for differential recognition of cell surface NS1 and protection in vivo, we mapped the epitopes of the anti-ZIKV NS1 mAbs. We localized NS1-binding sites critical for mAb recognition using (i) alanine-scanning mutagenesis and (ii) structure-guided charge-reversal mutagenesis^47^.

**Fig. 4:**
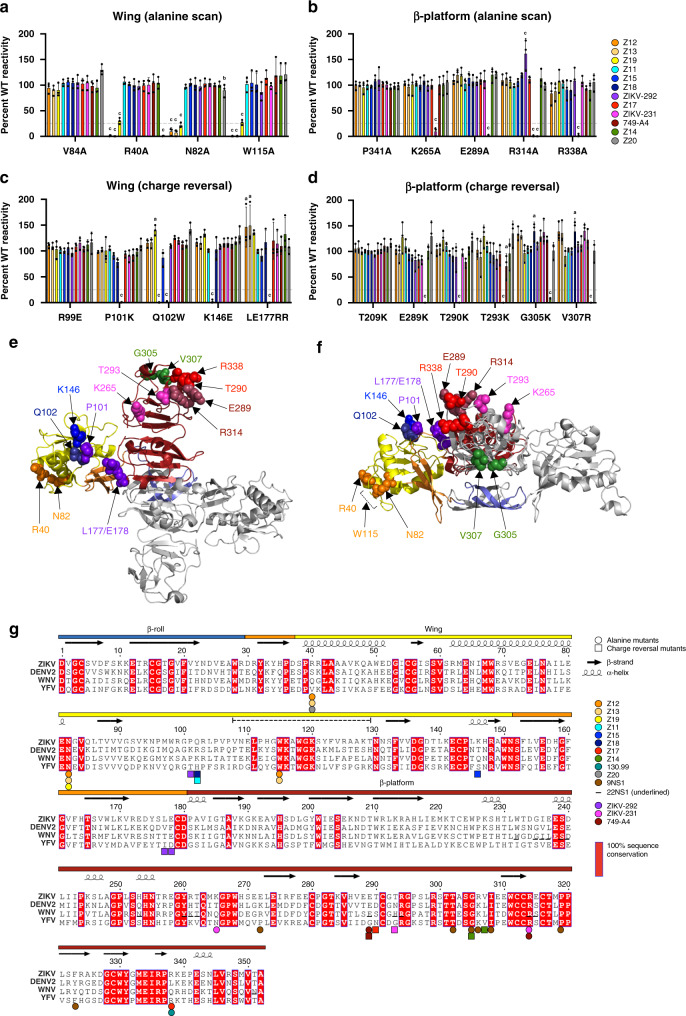
Epitope mapping of anti-ZIKV NS1 mAbs. Critical binding residues were mapped using **a**, **b** an alanine-scanning library and **c**, **d** targeted charge-reversal mutants. 293T cells were transfected with plasmids encoding ZIKV NS1 and the mAb reactivity to each mutant relative to WT NS1 was measured by flow cytometry. For each mutant, the relative mAb reactivity was normalized to the relative reactivity of an oligoclonal staining cocktail. Critical residues were defined as those mutants with <25% binding compared to WT NS1. Data are shown for critical residues in the wing (**a**, **c**) and β-platform (**b**, **d**) domains. A minimum of 5000 cells was collected for each sample. Data are the average of three experiments (error bars represent the mean ± SD) and were analyzed by two-way ANOVA with Holm–Sidak’s multiple comparison of each mutant to **a** V84A, **b** P341A, **c** R99E, or **d** T209K. Superscript letters indicate significance: a, *p* < 0.01; b, *p* < 0.001; c, *p* < 0.0001 (Z12: LE177/178RR, *p* = 0.009; Z13: LE177/178RR, *p* = 0.002; Z18: G305K, *p* = 0.007; V307R, *p* = 0.007; Z19: Q102W, *p* = 0.007; Z20: N82A, *p* = 0.0002; 749-A4: T293K, *p* = 0.0068). **e**, **f** Mapping of critical residues for anti-NS1 mAbs onto the crystal structure of the ZIKV NS1 dimer (PDB 5K6K) in top view (**e**) or side view (**f**). Critical residues are indicated by spheres and are color-coded according to mAb panel in **a**–**d**. In each structure, one monomer is gray and one is color-coded by domain (blue, β-roll; yellow, wing; red, β-platform; orange, connector subdomain and greasy finger). This figure was prepared using PyMOL. **g** Secondary structure representation of epitopes in ZIKV NS1 as defined by alanine-scanning (circles) and charge-reversal mutagenesis (squares). Sequence alignment is shown for ZIKV (H/PF/2013), DENV2 (Thailand/16681/84), WNV (NY99), and YFV (17D); red shading indicates 100% sequence conservation. Colored bars above the alignment indicate domains: β-roll (blue, 1–29); wing (yellow, 30–180) with connector subdomain (orange); β-platform (red, 181–352). The dashed line above the alignment indicates the wing flexible loop (108–129). The corresponding contact residues of 22NS1, an anti-WNV NS1 mAb, which were determined by X-ray crystallography^30^, are shown underlined in the alignment for comparison. This figure was prepared using ESPript 3.0^79^. Source data are provided as a Source Data file for **a**–**d**.

“Shotgun mutagenesis”^47^ was performed by substituting each residue of NS1 with alanine. Pre-existing alanine residues were substituted with serine and the invariant cysteine residues were not changed given their requirement in NS1 folding^31^. We assessed binding of anti-ZIKV NS1 mAbs to each mutant NS1 protein using a flow cytometric assay that compared staining of wild-type and mutant plasmid-transfected cells. Residues were deemed critical for mAb binding if a substitution resulted in <25% binding relative to that of WT NS1 but did not affect recognition by an oligoclonal pool of nine anti-NS1 mAbs. After evaluating the library of 340 mutants (Supplementary Data 1), we found that alanine substitution at three residues (R40, N82, and W115) within or structurally proximal to the wing domain flexible loop (residues 108–129) caused loss-of-binding of the non-protective group A mAbs, Z12 and Z13 (Fig. 4a). Sequencing of the variable heavy and light-chain regions revealed these two mAbs are related clonally, consistent with their shared binding patterns (Supplementary Table 4). The group A mAb Z19 also showed loss-of-binding with alanine substitutions at residues R40, N82, and W115, and mutation at residue R40 resulted in diminished binding of the non-protective group E mAb Z20. The protective group C mAbs mapped to residues on the exposed loop face of the β-platform domain (residues 181–352) (Fig. 4b). Human mAb ZIKV-231 mapped to residue K265 within the spaghetti loop (residues 219–272) and to residue R314 at the C-terminal tip of the β-platform domain (278–352), whereas Z17 and 749-A4 mapped to residues exclusively at the C-terminal tip of the β-platform (e.g., E289 and R338). For unclear reasons, we were unable to map the epitopes of the protective group B and non-protective group D mAbs using this approach.

A previous study identified immunodominant B-cell epitopes in DENV NS1 for mice and humans^48^. Using these epitope regions as guides, we engineered charge-reversal mutants (Supplementary Table 5) at solvent-exposed and adjacent residues predicted by the ZIKV NS1 atomic model^31^. We examined binding to these mutants as an alternative approach for mapping the anti-ZIKV NS1 mAbs in competition groups B (Z11, Z15, Z18, and ZIKV-292) and D (Z14). The protective group B murine mAbs mapped to residues clustered on the membrane-distal, charged surface of the wing domain: Z15: K146; Z11 and Z18: Q102; and ZIKV-292: P101 and L177/E178 (Fig. 4c). The non-protective group D mAb, Z14, mapped to two residues (G305 and V307) on the membrane-facing surface of the β-platform domain (Fig. 4d). This approach also identified key residues for binding of group C mAbs: Z17: T290; ZIKV-231: T293 (Fig. 4d and Supplementary Table 5).

Mutated residues that resulted in loss of Ab binding were mapped onto the ZIKV NS1 crystal (Fig. 4e, top view; Fig. 4f, side view) and secondary structures (Fig. 4g). The mAb competition groups localize to distinct epitopes. The non-protective mAbs (groups A, D, and E) map to the membrane-facing surface of the dimer including the wing flexible loop and β-strand face of the β-platform. The protective mAbs (groups B and C) map to the membrane-distal, accessible surface of the dimer including the charged surface of the wing domain and the loop face of the β-platform.

## Discussion

We examined the protective efficacy of newly generated mAbs targeting ZIKV NS1 in vivo. We identified four murine (Z11, Z15, Z18, and Z17) and three human mAbs (ZIKV-292, ZIKV-231, and 749-A4), which conferred protection against ZIKV in non-pregnant hSTAT2 KI mice, whereas five other murine mAbs (Z12, Z13, Z14, Z16, and Z20) did not show this activity. Three of the mAbs tested (Z17, ZIKV-292, and 749-A4) also protected against lethal ZIKV challenge in immunocompromised mice and reduced viral infection in the fetuses of pregnant mice. Whereas all mAbs recognize the soluble NS1 hexamer relatively equivalently, the protective and non-protective groups of antibodies differentially bind to cell surface NS1, which is believed to be expressed as a dimer^29,49,50^. The protective mAbs bind avidly and at high site density to cell surface NS1 and map to two accessible regions on the membrane-distal surface of the dimer: the wing domain and the loop face of the β-platform. In contrast, the non-protective mAbs bind relatively poorly to cell surface NS1 and map to less accessible epitopes near the hydrophobic, membrane-facing surface of the NS1 dimer.

A prior study suggested that protection against lethal WNV infection by anti-NS1 mAbs correlated with recognition of NS1 on the surface of infected cells^51^. Our data suggest that avid and high-density binding to cell surface NS1 is a determinant of protection for anti-NS1 antibodies. Structural studies suggest that dimeric NS1 interacts with the cell membrane through the hydrophobic β-roll domain, the flexible loop, and greasy finger of the wing domain^29,31^. These regions on the cell surface form of NS1 may be relatively inaccessible for antibody binding, which could explain why group A mAbs (Z12 and Z13; flexible loop) bound poorly and do not protect. The membrane-distal surfaces of the dimer, which should be accessible on cell surface forms of NS1, include the charged surface of the wing domain and the loop face of the β-platform domain. These regions are recognized efficiently by group B (wing) and C (loop face) mAbs, which protect against ZIKV infection in vivo.

The protective group C mAbs (Z17, ZIKV-231, and 749-A4) map to residues within the C-terminal region of the β-platform, which contains conserved sequences in flaviviruses. One epitope within the β-platform of NS1 has been described structurally. The WNV NS1-specific mAb, 22NS1, which protects against WNV challenge, contacts residues within an elongated loop that connects the fourth and fifth β-strand of the β-platform (spaghetti loop) as well as the C-terminal tip residues (Fig. 4g)^30^. Two of our group C mAbs bind epitopes that share contact residues with 22NS1. ZIKV-231 maps to two C-terminal tip residues, which are structurally equivalent to 22NS1 epitope contacts (T293 and R314), and the third ZIKV-231 residue we identified (K265) is located within the spaghetti loop proximal to the 22NS1 epitope. We also identified two C-terminal tip epitope residues for the broadly NS1 reactive 749-A4 mAb (E289 and R314), and both of these are conserved in WNV and contacted by 22NS1. Murine mAb 9NS1, which was isolated against WNV^35^, cross-reacts with all tested flavivirus NS1 proteins and also maps to conserved residues at the C-terminal tip. Thus, it is possible that other mAbs binding this C-terminal epitope could provide cross-protection against other flaviviruses, although this warrants further examination. Whereas we observed a protective effect against ZIKV following passive administration of group C mAbs, some anti-DENV NS1 antibodies that bind C-terminal epitopes (particularly residues 305–330) reportedly react with human endothelial cells, platelets, and plasminogen, and are speculated to contribute to pathogenesis^52–54^.

The wing domain, particularly within residues 101–130, contains immunodominant B-cell epitopes for DENV NS1 in mice and humans^48^. The group A (Z12, Z13, and Z19), group B (Z11, Z15, Z18, and ZIKV-292), and group E (Z20) mAbs map to these residues, although to distinct regions. The protective group B mAbs map to the membrane-distal, hydrophilic surface of the wing domain, whereas the non-protective group A mAbs map to the hydrophobic flexible loop (W115) of the wing domain. A previously generated murine mAb against WNV NS1, 16NS1, also maps to residues (W118 and I122) within the flexible loop^36^. The flexible loop (residues 108–129) was disordered in the WNV and DENV NS1 crystal structures, but stabilized in the ZIKV NS1 structure^31,32^. This region is believed to facilitate membrane association with the distal end of the wing domain via conserved hydrophobic residues. MAb 16NS1 protected mice against lethal WNV challenge^35^, whereas Z12 and Z13 failed to inhibit ZIKV infection. Although further studies with a larger panel of mAbs that map to this particular epitope are warranted, we speculate that the flexible wing domain hydrophobic loops of ZIKV and WNV NS1 may interact differently with membranes, thereby modulating their unique antibody epitope accessibilities. Moreover, structural studies that achieve atomic-level resolution of mAb bound to NS1 may help to identify further how protective and non-protective mAbs engage NS1.

We evaluated a combination of two mouse mAbs (Z15 and Z17) during pregnancy, to determine whether concurrent targeting of more than one epitope could enhance protection, as there is precedence for this approach in antiviral antibody therapies^55–57^. This combination trended toward reducing viral infection in fetal heads, although it did not attain statistical significance. We evaluated a second combination of human antibodies that bound distinct epitopes (ZIKV-292 and 749-A4) during pregnancy as well, but did not observe greater protection. In the future, additional two- or even three-antibody combinations could be tried in this model.

Passive transfer of NS1-specific mAbs can protect mice from lethal challenge with WNV, JEV, or YFV^34–36^. The mechanism of protection for many of the anti-WNV NS1 mAbs was Fc-dependent, as it was lost in mice lacking Fc-γ receptors or C1q^35^. Analogously, an anti-YFV NS1 antibody mediated antiviral protection in mice when using IgG2a, but not IgG1 isotype switch variants or cleaved F(ab′)_2_ fragments^34^. More recently, passive transfer of a single human anti-ZIKV NS1 mAb (AA12) protected *Stat2*^*−/−*^ mice against lethal challenge with ZIKV^37,38^, although epitope mapping data were not provided. Similar to our data, protection was Fc-dependent, as determined with loss-of-function mutations in the Fc region^58^. To test Fc-dependent protection by the human mAbs, we generated Fc region LALA variants, which have reduced binding to Fc-γ receptors and complement. Although we observed loss of protection in the hSTAT2 KI mice using the Fc region LALA variants, the effect might be greater with LALA-PG or other mutations that more completely eliminate binding to Fc receptors and complement^58^. Given that ZIKV-infected cells express high levels of NS1 on the plasma membrane, NS1-specific antibodies that bind avidly and with high site density may promote clearance of infected cells via cross-linking and promoting Fc-mediated effector functions. The specific effector functions of a mAb might explain differences we observed in protection between non-pregnant and pregnant mice. Mouse mAb Z17 protects in both mouse models, whereas Z15 protects only in non-pregnant mice. Given that Z17 induces more complement deposition than Z15 in Fc effector function assays, complement-dependent cytolysis or opsonization might contribute to antibody-mediated protection of the fetus against ZIKV. Indeed, human mAbs ZIKV-292 and 749-A4, which protected in pregnancy, also promoted complement deposition. We cannot exclude the possibility that antibodies also may protect by targeting the secreted form of NS1 and blocking its potential effects on immune evasion and pathogenesis^13,18,19,26–28^. Indeed, soluble ZIKV NS1 protein may promote placental dysfunction and permeability through modulation of glycosaminoglycan expression on chorionic villi^28^.

Most antibody-based therapeutic or vaccine strategies against ZIKV have focused on generating neutralizing antibodies against the viral envelope protein, which indeed protect in different animal models^40,59,60^. However, pre-existing anti-envelope protein antibodies reportedly can augment both ZIKV and DENV infection, a phenomenon termed ADE^61,62^. NS1-targeted vaccines have been developed as an alternative to envelope protein-targeted strategies to avoid ADE, because NS1 is absent from the virion. Indeed, NS1-based vaccines can protect mice against lethal YFV, TBEV, and ZIKV infections^63–67^. Whether any of these vaccine candidates can protect the developing fetus from ZIKV, however, remains to be tested. Given that several anti-NS1 mAbs limited ZIKV infection in the placenta and fetus, NS1-targeted vaccines or antibody-based therapies might confer protection during pregnancy, without the risk of immune enhancement of homologous or heterologous flavivirus infection.

In summary, we defined a panel of anti-NS1 mAbs that limit ZIKV infection in non-pregnant and pregnant mice and established functional correlates of protection: binding to cell surface forms of NS1 and engagement of Fc receptors by mAbs. One limitation of this study is that it does not assess protection in a model of ZIKV-induced fetal demise. To achieve such extensive disease in mice requires the use of highly immunocompromised animals where ZIKV disseminates widely and replicates to high titer^39,68^. Anti-NS1 mAbs could be developed alone or in combination with anti-envelope protein antibodies (the latter possibly engineered to lack Fc interactions to avoid ADE) for prevention against ZIKV infection and congenital disease. Our studies suggest that NS1-specific antibodies recognizing antigenic sites in the charged surface of the wing domain and C-terminal end of the β-platform domain loop face optimally mediate protection in vivo, because they are accessible in the cell surface-associated form of dimeric NS1. Vaccines targeting these epitopes may induce the most protective NS1-specific antibodies. Future studies that elucidate the atomic structures of protective mAbs bound to NS1 may refine antigen engineering strategies for rational NS1 vaccine design.

## Methods

### Ethics statement

All animal procedures were carried out in accordance with the recommendations in the Guide for the Care and Use of Laboratory Animals of the National Institutes of Health. The protocols were approved by the Institutional Animal Care and Use Committee at the Washington University School of Medicine (assurance number A3381-01). To minimize animal discomfort and suffering, injections were performed under anesthesia that was induced and maintained with ketamine hydrochloride and xylazine. All human anti-ZIKV mAbs, except for 749-A4, were isolated from the blood of subject 973^40,69^, who had a history of ZIKV (African lineage strain) infection. Blood samples were collected by the Vanderbilt Clinical Trials Center following written informed consent using a protocol approved by the Vanderbilt University Medical Center Institutional Review Board. Human mAb 749-A4 was isolated after written informed consent, from blood collected from an in-patient who tested positive for DENV by reverse-transcriptase PCR (RT-PCR) at the Hospital for Tropical Diseases^70^. The study protocol was approved by the Scientific and Ethical Committee of the Hospital for Tropical Diseases, the Oxford Tropical Research Ethical Committee, and the Riverside Ethics Committee in the UK.

### Cells

Vero, HEK293T, and C6/36 cells (all from American Type Culture Collection, ATCC) were propagated in Dulbecco’s modified Eagle Medium (DMEM) supplemented with 10% fetal bovine serum (FBS), 1 mM sodium pyruvate, and 10 mM HEPES, pH 7.3. Hybridoma cells were grown in Isocove’s modified Eagle Medium supplemented with 20% FBS (Hyclone), 1 mM sodium pyruvate, and 100 U/mL of penicillin and streptomycin.

### Viruses

The mouse-adapted variant of ZIKV strain Dakar 41525 (Senegal, 1984) has been described^39^. Virus stocks were propagated in C6/36 or Vero cells and titered by focus-forming assay^71^.

### Purification of NS1 domain (D)II/III protein

A ZIKV NS1 C-terminal construct was engineered, expressed, and purified essentially as described for WNV and DENV NS1^30^. Briefly, residues 966–1148 of the ZIKV strain H/PF/2013 polyprotein (residues 172–352 of ZIKV NS1) were cloned into the NheI and NotI restriction sites of pET21a for expression in BL21 (DE3) codon plus *Escherichia coli* cells by autoinduction^72^. Inclusion bodies (200–300 mg) were denatured in 7 M guanidinium hydrochloride and 30 mM β-mercapthanol for 1 h at 37 °C, centrifuged for 10 min at 4 °C, and diluted to 2 M guanidinium hydrocloride in 50 mM sodium acetate pH 5.2. The supernatant was filtered and refolded by rapid, serial dilution (1 mL injections, hourly) in 1 L of refolding buffer (400 mM l-arginine, 100 mM Tris-base pH 8.3, 2 mM EDTA, 0.5 mM oxidized glutathione, 5 mM reduced glutathione, and 0.2 mM phenylmethanesulfonyl fluoride) at 4 °C. The recombinant protein was concentrated using a stirred cell concentrator with YM10 membrane (Millipore), centrifuged to remove aggregates, and purified on a S-75 size-exclusion chromatography column equilibrated in 20 mM HEPES pH 7.4, 150 mM NaCl, and 0.01% NaN_3_.

### Antibody generation

(i) Mouse mAbs: Six-week-old female BALB/c mice were primed and boosted 3 weeks later with 10^6^ focus-forming units (FFU) of ZIKV following administration of 1 mg of an Ifnar1-blocking mAb (clone MAR1-5A3; Leinco I-401)^73^. At 6 weeks after initial infection, mice were boosted with 25 μg of recombinant ZIKV NS1 protein (Native Antigen) mixed 1 : 1 with Freund’s Incomplete Adjuvant. Mice were given a final pre-fusion boost with 25 μg of ZIKV NS1 protein in phosphate-buffered saline (PBS) at 10 weeks after initial infection. Three days later, splenocytes were collected and fused with P3X63 Ag8.653 myeloma cells to generate hybridomas^74^. Supernatants were screened for antibody binding to ZIKV-infected cells by flow cytometry or to recombinant ZIKV NS1 protein by ELISA. Briefly, ZIKV-infected C6/36 cells (multiplicity of infection (MOI) of 0.1, 3 dpi) were fixed with 4% paraformaldehyde (PFA), permeabilized in PBS, 0.1% saponin, 0.1% bovine serum albumin (BSA), and then incubated with hybridoma culture supernatants supplemented with 0.1% saponin. Anti-NS1 mAbs were detected using Alexa Fluor 647-conjugated goat anti-mouse IgG (1 : 2000 dilution; Thermo Fisher). For screening by direct ELISA, recombinant ZIKV NS1 (0.4 μg/ml; Native Antigen) was immobilized onto MaxiSorp 96-well plates (Thermo Fisher) overnight at 4 °C in 50 μl of sodium bicarbonate buffer, pH 9.3. Subsequently, plates were washed four times with PBS and blocked with ELISA buffer (PBS, 1% BSA, and 0.05% Tween 20) for 1 h at 37 °C. Plates then were incubated with undiluted hybridoma culture supernatants for 1 h at room temperature. After washing four times with ELISA buffer, plates were incubated with biotinylated anti-mouse IgG (H + L; 1 : 2000 dilution; Jackson ImmunoResearch) for 30 min. Plates were washed again and incubated with streptavidin-conjugated horseradish peroxidase (1 : 625 dilution; Vector Laboratories) for 30 min. After a final wash series, plates were developed using 3,3’,5,5’-tetramethylbenzidine substrate (Agilent). The reaction was stopped using 2 N H_2_SO_4_ and absorbance at 450 nm was read with a TriStar Microplate Reader (Berthold Technologies) to detect anti-NS1 antibodies. Positive hybridomas were cloned by limiting dilution and selected mAbs were purified by a commercial source (Bio X Cell). (ii) Human mAbs: Human mAbs were generated two ways as follows: (a) MAbs ZIKV-231 and ZIKV-292: the ZIKV-immune human donors were described previously^40,69^. Peripheral blood mononuclear cells (PBMCs) from heparinized blood were isolated using Ficoll-Histopaque and density gradient centrifugation. Ten million PBMCs were cultured in 384-well plates (Nunc) using culture medium (ClonaCell-HY medium A; StemCell Technologies) supplemented with 8 μg/mL of the TLR agonist CpG (phosphorothioate-modified oligodeoxynucleotide ZOEZOEZZZZZOEEZOEZZZT; Invitrogen), 3 μg/mL of Chk2 inhibitor (Sigma), 1 μg/mL of cyclosporine (Sigma), and clarified supernatants from cultures of B95.8 cells (ATCC) containing Epstein–Barr virus. After 7 days, cells from each 384-well culture plate were expanded into four 96-well culture plates (Falcon) using ClonaCell-HY medium A containing 8 μg/mL of CpG, 3 μg/mL of Chk2 inhibitor, and 10^7^ irradiated heterologous human PBMCs (Nashville Red Cross), and cultured for an additional 4 days. Supernatants were screened by ELISA for reactivity with ZIKV NS1. Hybridoma cell lines were cloned by single-cell flow cytometric sorting in a sterile FACSAria III cytometer (BD Biosciences). (b) MAb 749-A4: 749-A4 was generated from plasmablasts during acute DENV infection^70,75^. PBMCs were stained with anti-CD3 (1 : 20; BD Pharmingen 345766), anti-CD19 (1 : 50; Dako R0808), anti-CD20 (1 : 20; BD Pharmingen 345794), anti-CD27 (1 : 20; BD Pharmingen 555440), anti-CD38 (1 : 20; BD Pharmingen 555462), and plasmablasts were single-cell sorted into 96-well plates containing RNase inhibitor (Promega) by gating on CD19^+^, CD3^−^, CD20^lo^ to CD20^−^, CD27^hi^, and CD38^hi^ by RT-PCR (Qiagen). Nested PCR (Qiagen) was performed to amplify genes encoding γ-chain, λ-chain, and κ-chain with “cocktails” of primers specific for human IgG (Supplementary Data 2)^75^. PCR products encoding heavy- and light-chain genes were digested with the appropriate restriction endonuclease(s) and cloned into expression vectors for human IgG1 or Ig κ-chain or λ-chain (gift from H. Wardemann)^76^. For the expression of antibodies, plasmids encoding heavy and light chains were co-transfected into 293T cells using the polyethylenimine method^77^.

### Mouse experiments

Most  antibody protection studies were performed using hSTAT2 KI mice^39^. For non-pregnancy studies, 3- to 4-week-old male and female hSTAT2 KI mice were administered 200 μg of mAb (anti-NS1 or isotype control mAb) via intraperitoneal injection and then immediately inoculated subcutaneously in the footpad with 10^5^ FFU of ZIKV in a 50 μL volume. Serum samples were obtained at 3 dpi via facial vein puncture and tissues were collected at 9 dpi following perfusion with 20 mL of PBS. For pregnancy studies, 8- to 16-week-old female hSTAT2 KI mice were paired with male hSTAT2 KI mice and checked each morning for a copulation plug; this day was defined as E0.5. On E6.5, plugged mice were administered 250 μg of mAb (anti-NS1 or isotype control mAb) via intraperitoneal injection and then immediately inoculated subcutaneously in the footpad with 10^6^ FFU of ZIKV in a 50 μL volume. Mice were euthanized on E13.5 and the maternal spleen, placentas, and fetal heads were collected. For antibody cocktail treatments, mice received 125 μg of each antibody.

To assess antibody-mediated protection against lethal ZIKV infection, 4- to 5-week-old C57BL/6J mice were administered 1 mg of anti-Ifnar1-blocking mAb (MAR1-5A3) and 500 μg of anti-NS1 or isotype control mAb via intraperitoneal injection^39^. The following day, mice were inoculated subcutaneously in the footpad with 10^3^ FFU of ZIKV in a 50 µL volume. Lethality and weights were tracked for 21 days, at which point surviving mice were euthanized.

### Viral burden analysis

Organs were weighed and homogenized by bead dissociation using a MagNA Lyzer (Roche) in a volume of DMEM containing 2% FBS. Viral RNA was isolated from tissue homogenates using the RNeasy Mini kit (Qiagen), as per the manufacturer’s instructions. ZIKV RNA levels were determined by TaqMan one-step quantitative RT-PCR (forward primer: 5′-TTCGGACAGCCGTTGTCCAACACAAG-3′; reverse primer: 5′-CCACCAATGTTCTCTTGCAGACATATTG-3′; probe: 5′-56-FAM/AGCCTACCT/ZEN/TGACAAGCAGTC/3IABkFQ-3′)^39^. To calculate FFU equivalents from RNA levels, an RNA standard curve was generated from a defined viral stock.

### Antibody domain mapping and cross-reactivity analysis

Recombinant NS1 proteins (0.4 μg/mL) were immobilized onto MaxiSorp 96-well plates (Thermo Fisher) overnight in 50 μL of sodium bicarbonate buffer, pH 9.3. For mAb domain mapping, full-length ZIKV NS1 (residues 1–352) or ZIKV NS1 DII/III (residues 172–352)^35^ was immobilized. For determination of cross-reactivity, ZIKV, DENV2, WNV, JEV, TBEV, or YFV NS1 proteins (all from Native Antigen) were adsorbed to wells of MaxiSorp microtiter plates overnight at 4 °C. Subsequently, plates were washed four times with PBS and blocked with ELISA buffer (PBS, 1% BSA, and 0.05% Tween 20) for 1 h at 37 °C. Plates then were incubated with anti-NS1 or isotype control mAbs diluted in ELISA buffer for 1 h at room temperature. After washing four times with ELISA buffer, plates were incubated with biotinylated goat anti-human or goat anti-mouse IgG (H + L; 1:2000 dilution; Jackson ImmunoResearch) for 30 min. Plates were washed again and incubated with streptavidin-conjugated horseradish peroxidase (1:625 dilution; Vector Laboratories) for 30 min. After a final wash series, plates were developed using 3,3′,5,5′-tetramethylbenzidine substrate (Agilent). The reaction was stopped using 2 N H_2_SO_4_ and absorbance at 450 nm was read with a TriStar Microplate Reader (Berthold Technologies).

### Antibody binding to NS1 on the cell surface

Vero cells were inoculated with ZIKV at an MOI of 1. After 24 h, the cells were washed in PBS, detached after incubation in 10 mM EDTA in PBS for 15 min at 37 °C, and then washed again in chilled PBS, 4 mM EDTA, 0.4% BSA (fluorescence-activated cell sorting (FACS) buffer). The cells then were incubated with serial dilutions (20 μg/mL to 2 pg/mL) of each anti-NS1 or an isotype control mAb for 1 h at 4 °C. After washing in FACS buffer, cells were stained with Fixable Viability Dye eFluor 506 (eBioscience) and Alexa Fluor 647 conjugated to goat anti-human or anti-mouse IgG (Thermo Fisher). Cells were washed before fixing in 4% PFA in PBS for 10 min and then processed on a MACSQuant Analyzer (Miltenyi Biotec) using FlowJo software. After gating on live cells, the percent reactivity to cell surface-expressed NS1 was determined and plotted for each dilution of mAb (Supplementary Fig. 2a). The EC_50_ of binding value was calculated using a 4-parameter logistic curve.

### Antibody competition ELISA

Anti-NS1 mAbs were conjugated to biotin using a sulfo-NHS-biotin kit (Thermo Scientific). MaxiSorp 96-well plates were coated with ZIKV NS1 protein, washed, and blocked as described above. Plates then were incubated with unmodified anti-NS1 mAbs at 10 μg/mL for 1 h at room temperature. Without washing, biotinylated mAbs were added to the plates at pre-optimized concentrations and incubated for 10 min at room temperature. Plates then were washed four times in PBS, 0.05% Tween 20, and incubated with streptavidin-conjugated horseradish peroxidase (1 : 625 dilution) for 30 min. After washing, plates were developed and absorbance was read as described above.

### FcγR I ELISA

MaxiSorp 96-well plates were coated overnight with 50 μL of recombinant human FcγR I (CD64) protein (1 μg/mL; R&D Systems), washed, and blocked as described above. Serial dilutions of the anti-NS1 human antibodies (WT and LALA variants) in ELISA buffer were added to the plates and incubated for 1 h at room temperature. Subsequently, plates were washed four times and incubated with biotin-conjugated goat anti-human IgG (H + L; 1 : 2000 dilution; Thermo Fisher) for 45 min. Plates then were washed and incubated with streptavidin-conjugated horseradish peroxidase (1 : 625 dilution) for 30 min. After a final wash series, plates were developed and absorbance was read as described above.

### Alanine-scanning mutagenesis and epitope mapping

Epitope mapping was performed using alanine-scanning mutagenesis^47^. A mammalian expression vector (pFM-A1.2) was constructed to encode full-length ZIKV NS1 (strain H/PF/2013), preceded by the native signal sequence (last 25 amino acids of envelope protein) and followed in frame with a hexahistidine affinity tag. The pFM-A1.2 expression vector was subjected to site-directed mutagenesis (GENEWIZ) to generate a library of 340 total mutants. Non-alanine codons were mutated to alanine, alanine codons were mutated to serine, and cysteine codons were not changed. HEK293T cells were transfected with each mutant construct by using Lipofectamine 3000 (Thermo Fisher) and incubated at 37 °C for 1 day to allow for protein expression. Cells were fixed, permeabilized, and incubated with individual mAbs or an oligoclonal cocktail of anti-NS1 mAbs (Z2, Z4, Z11, Z12, Z14, Z15, Z17, Z18, 130.99) as a control for NS1 protein expression. Data for binding of mouse mAb 130.99 to recombinant and cell surface forms of NS1 is in Supplementary Fig. 3 and its competition ELISA data are included in Supplementary Table 3. Anti-NS1 mAb binding was detected using Alexa Fluor 647 conjugated to goat anti-human or goat anti-mouse IgG (1 : 2000 dilution; Thermo Fisher). Flow cytometry was performed on a MACSQuant Analyzer (Miltenyi Biotec) and analyzed using FlowJo software (Supplementary Fig. 2b). Based on previously published criteria^78^, critical binding residues for each mAb were defined as alanine mutants with <25% reactivity relative to WT protein. Mutants with <70% binding (compared to WT) of the oligoclonal antibody pool were considered poorly expressed and excluded from further analysis. Additional structure-guided charge-reversal mutants were generated (GENEWIZ) and assessed for binding to individual mAbs or the oligoclonal cocktail. The following mAbs were mapped using the entire alanine-scanning library and all charge-reversal mutants: Z12, Z13, Z14, Z15, Z17, Z18, ZIKV-231, ZIKV-292, and 749-A4. The other mAbs (Z11, Z19, and Z20) were mapped using the mutants identified as part of epitopes for the former set of mAbs.

### Antibody-dependent complement deposition

Recombinant ZIKV NS1 protein (Native Antigen) was biotinylated and coupled to red fluorescent Neutravidin beads (Life Technologies) and then incubated with serial dilutions of anti-NS1 mAbs for 2 h at 37 °C to allow for binding. Freshly reconstituted guinea pig complement (Cedarlane Labs) was diluted 1 : 10 in veronal buffer containing 0.1% gelatin (Boston Bioproducts), which then was incubated with the antibody-bead complexes for 20 min at 37 °C. After washing in 15 mM EDTA in PBS, the complexes were stained with a fluorescein isothiocyanate (FITC)-conjugated anti-guinea pig C3b antibody (1 : 100; MP Biomedicals 0855385) for 15 min at room temperature. Complement deposition was detected using an IntelliCyt iQue Screener Plus or a 3 L Stratedigm S1300EXI flow cytometer, and the median fluorescent intensity of FITC was measured for all beads using FlowJo software.

### Antibody-dependent neutrophil and cellular phagocytosis

Recombinant ZIKV NS1 protein (Native Antigen) was biotinylated and conjugated to streptavidin-coated Alexa Fluor 488 beads. NS1-coated beads were incubated with serial dilutions of mAbs (5–0.0016 μg/mL) in cell culture medium for 2 h at 37 °C. For the neutrophil phagocytosis assays, bone marrow cells from C57BL/6 mice were collected, washed with PBS, and aliquoted into 96-well plates (5.0 × 10^4^ cells per well). The bead–antibody complexes were added to cells and incubated for 1 h at 37 °C. After washing, cells were stained with the following antibodies: CD11b APC (1:20; clone M1/70; BioLegend 101212), CD11c APC/Cy7 (1:20; clone N418; BioLegend 117324), Ly6G Pacific Blue (1:20; clone 1A8; BioLegend 127612), Ly6C BV605 (1 : 20; clone HK1.4; BioLegend 128036), and CD3 PE/Cy7 (1:20; clone 17A2; BioLegend 100220). Cells were fixed with 4% PFA, processed on an IntelliCyt iQue Screener Plus flow cytometer, and analyzed using FlowJo software. Neutrophils were defined as CD3^−^ and CD11c^−^ cells that were Ly6C^−^, CD11b^+^, and Ly6G^+^ (Supplementary Fig. 2c). The neutrophil phagocytosis score was determined using the following calculation: (% Alexa Fluor 488^+^ cells) × (geometric mean fluorescent intensity of Alexa Fluor 488^+^ cells)/10,000.

For the cellular phagocytosis assays, J774A.1 (ATCC TIB-67) murine monocytic cells were incubated with the NS1-coated bead–antibody complexes for 1 h 37 °C. Cells were washed in 5 mM EDTA PBS, fixed with 4% PFA, and analyzed on an IntelliCyt iQue Screener Plus or a 3 L Stratedigm S1300EXI flow cytometer. The cellular phagocytic score was calculated as described for the neutrophil phagocytosis assays.

### Statistical analysis

The statistical tests for each data set are indicated in the respective legends and were performed using GraphPad Prism software. Viral burden data were analyzed by a Kruskal–Wallis test with Dunn’s post test, survival analyses were performed using a Mantel–Cox log-rank test with Bonferroni correction, weight loss data were analyzed by two-way analysis of variance (ANOVA) with Dunnett’s post test, and epitope mapping data were analyzed by two-way ANOVA with Holm–Sidak’s post test. Statistical significance was defined as *p* < 0.05.

### Reporting summary

Further information on research design is available in the Nature Research Reporting Summary linked to this article.

## Supplementary information

Supplementary Information

Description of Additional Supplementary Files

Supplementary Data 1

Supplementary Data 2

Reporting Summary

## Data Availability

The authors declare that all data supporting the findings of this study are available within the paper and its Supplementary Information, or from the corresponding author upon request. Hybridomas or purified antibodies will be made available under an MTA with Washington University or Vanderbilt University.  Source data are provided with this paper.

## Source data

Source Data
