# Supplementary material for: Antibodies targeting epitopes on the cell-surface form of NS1 protect against Zika virus infection during pregnancy
Source: Nat Commun. 2020 Oct 19;11:5278. doi: 10.1038/s41467-020-19096-y (PMC7572419; doi:10.1038/s41467-020-19096-y)
Supplement: Supplementary file 1 — Supplementary Information [file 41467_2020_19096_MOESM1_ESM.pdf]

SUPPLEMENTARY FIGURES AND TABLES

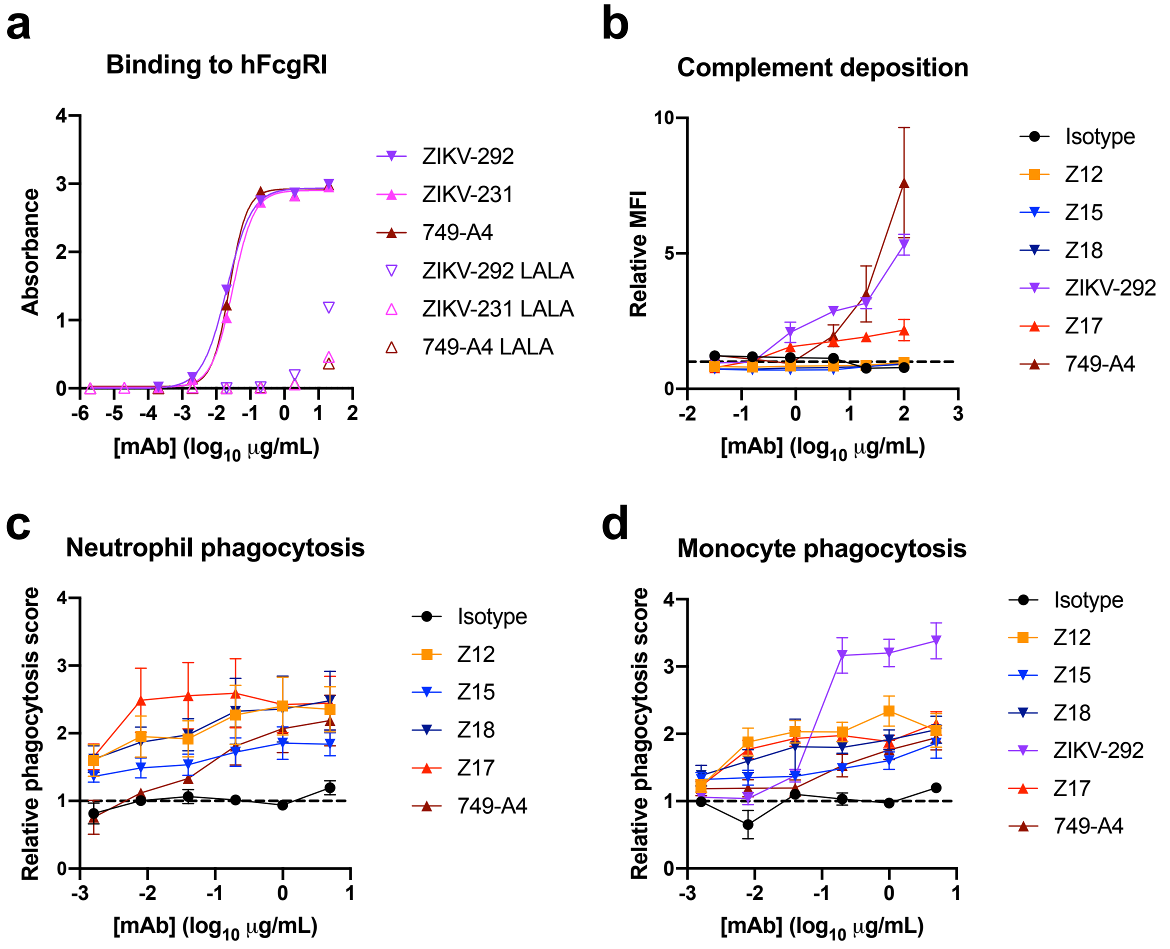

Supplementary Figure 1. Effector functions of anti-NS1 mAbs. (**a**) Binding to hFcγRI (CD64) of wild-type and LALA variant mAbs was assessed by ELISA. Absorbance values are the average of duplicates and representative of 2 experiments. (**b**) Complement deposition assay. ZIKV NS1 protein was coupled to beads and incubated with anti-NS1 or isotype control mAb at indicated concentrations. Following incubation with complement, C3b deposition was assessed by flow cytometry (a minimum of 15,000 beads were collected). Data are presented as the mean of 2 experiments performed in duplicate or triplicate and represent the fold increase in mean fluorescent intensity (MFI) of C3b binding for anti-NS1 mAbs compared to isotype control mAb (dashed line); error bars represent SD. (**c-d**) Antibody-dependent neutrophil (**c**) and monocyte (**d**) phagocytosis of ZIKV NS1-coated fluorescent beads. The phagocytosis score was calculated as described in the Methods and is shown as the fold over the isotype control. A minimum of 7000 (**c**) or 2000 cells (**d**) were collected for each sample. Data are presented as the mean of 2 experiments performed in duplicate or triplicate; error bars represent SD. (**a-d**) Source data are provided as a Source Data file.

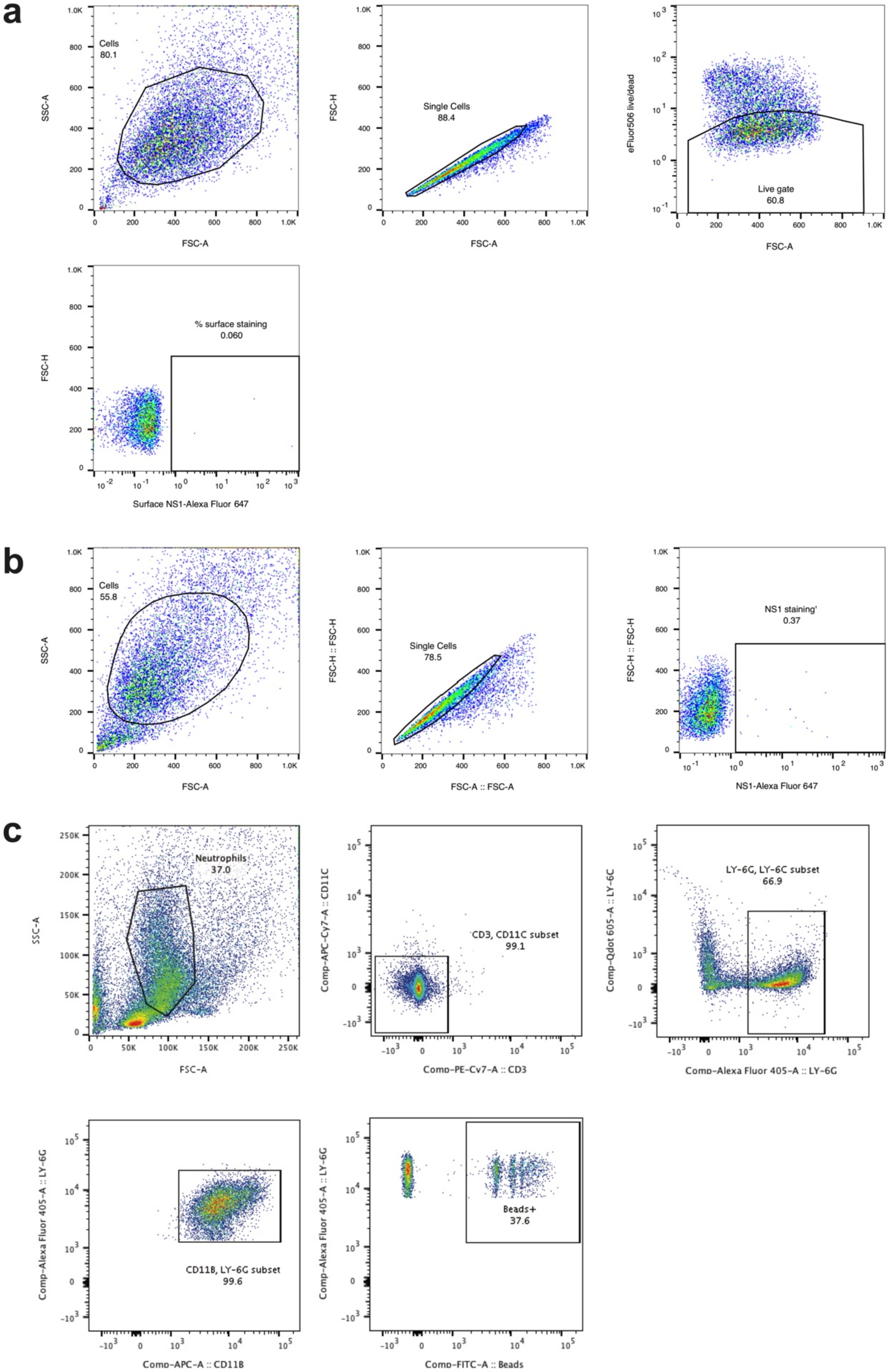
a
Cells
80.1
SSC-A
FSC-A
Single Cells
88.4
FSC-H
FSC-A
eFluor506 live/dead
Live gate
60.8
FSC-A
% surface staining
0.060
FSC-H
Surface NS1-Alexa Fluor 647
b
Cells
55.8
SSC-A
FSC-A
Single Cells
78.5
FSC-H :: FSC-H
FSC-A :: FSC-A
NS1 staining'
0.37
FSC-H :: FSC-H
NS1-Alexa Fluor 647
c
Neutrophils
37.0
SSC-A
FSC-A
CD3, CD11C subset
99.1
Comp-APC-Cy7-A :: CD11C
Comp-PE-Cy7-A :: CD3
LY-6G, LY-6C subset
66.9
Comp-Qdot 605-A :: LY-6C
Comp-Alexa Fluor 405-A :: LY-6G
CD11B, LY-6G subset
99.6
Comp-Alexa Fluor 405-A :: LY-6G
Comp-APC-A :: CD11B
Beads+
37.6
Comp-Alexa Fluor 405-A :: LY-6G
Comp-FITC-A :: Beads

Supplementary Figure 2. **Gating strategy for flow cytometry experiments.** (**a**) Gating strategy related to **Figure 3**. ZIKV-infected Vero cells binding to anti-NS1 mAb were initially gated on live, single cells using a viability dye (eFluor506). Negative staining by an isotype control mAb is shown to demonstrate how the gate was set to quantify binding by anti-NS1 mAbs. (**b**) Gating strategy related to **Figure 4**. Transfected 293T cells were initially gated on single cells. Negative staining of mock-transfected cells by the oligoclonal cocktail is shown to demonstrate how the gate was set to quantify binding to WT and mutant NS1. (**c**) Gating strategy related to **Supplementary Figure 1b**. Neutrophils were defined as $CD3^-$ and $CD11c^-$ cells that were $Ly6C^-$, $CD11b^+$, and $Ly6G^+$.

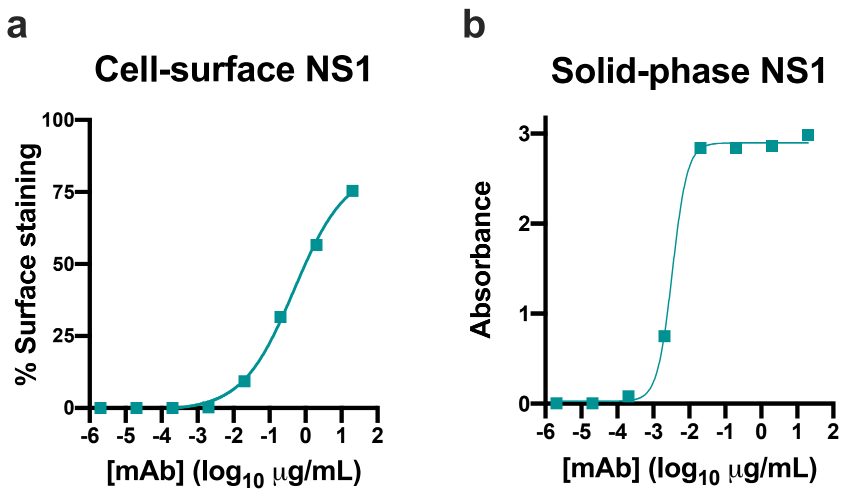

Supplementary Figure 3. Binding properties of anti-NS1 mouse mAb 130.99. Binding to different forms of NS1 was assessed for anti-NS1 mAb 130.99 (IgG1). (**a**) Binding to cell-surface-associated NS1 was assessed by flow cytometry following staining of live, ZIKV-infected Vero cells at 4°C with serial dilutions of mAb. The percentage of cells staining for each concentration of mAb is shown as the average of 2 replicates. (**b**) Binding of mAb 130.99 to recombinant, soluble NS1 was assessed by direct ELISA. Absorbance values for each concentration of mAb are the average of duplicates. Source data are provided as a Source Data file.

Supplementary Table 1. List of anti-NS1 mAbs

mAb	Species[a]	Isotype[b]	Binding to recombinant NS1[b]	Binding to infected cells[c]
ZIKV-198	Human	IgG1	+	+
ZIKV-231	Human	IgG1	+	+
ZIKV-240	Human	IgG1	+	+
ZIKV-243	Human	IgG1	+	+
ZIKV-267	Human	IgG1	+	+
ZIKV-276	Human	IgG1	+	+
ZIKV-277	Human	IgG1	+	+
ZIKV-283	Human	IgG1	+	+
ZIKV-292	Human	IgG1	+	+
ZIKV-303	Human	IgG1	+	+
ZIKV-307	Human	IgG3	+	+
ZIKV-315	Human	IgG1	+	+
ZIKV-318	Human	IgG1	+	+
ZIKV-327	Human	IgG1	+	+
ZIKV-332	Human	IgG1	+	+
ZIKV-344	Human	IgG1	+	+
ZIKV-346	Human	IgG1	+	+
ZIKV-352	Human	IgG1	+	+
ZIKV-354	Human	IgG1	+	+
ZIKV-363	Human	IgG1	+	+
ZIKV-364	Human	IgG1	+	+
ZIKV-379	Human	IgG1	+	+
749-A4	Human	IgG1	+	+
13G3	Mouse	IgG1	+	+
33E7	Mouse	IgG1	+	+
5B8	Mouse	IgG1	+	+
7A3	Mouse	IgG1	+	+
22C8	Mouse	IgG1	+	+
22E4	Mouse	IgG1	+	+
25F8	Mouse	IgG1	+	+
3B5	Mouse	IgG1	+	+
15E4	Mouse	IgG1	+	+
22G3	Mouse	IgG1	+	+
22G10	Mouse	IgG1	+	+
34E4	Mouse	IgG1	+	+
39B6	Mouse	IgG1	+	+
4D7	Mouse	IgG1	+	+
4F3	Mouse	IgG1	+	+
8E1	Mouse	IgG1	+	+
21H10	Mouse	IgG1	+	+
19A11	Mouse	IgG1	+	+
33E8	Mouse	IgG1	+	+
5G6	Mouse	IgG1	+	+
39A6	Mouse	IgG1	+	+
39C6	Mouse	IgG1	+	+

20B7	Mouse	IgG1	+	+
Z11	Mouse	IgG2a	+	+
Z12	Mouse	IgG2a	+	+
Z13	Mouse	IgG2a	+	+
Z14	Mouse	IgG2a	+	+
Z15	Mouse	IgG2a	+	+
Z16	Mouse	IgG2a	+	+
Z17	Mouse	IgG2a	+	+
Z18	Mouse	IgG2a	+	+
Z19	Mouse	IgG2a	+	+
Z20	Mouse	IgG2a	+	+
Z21	Mouse	IgG2a	+	+
Z22	Mouse	IgG2b	+	+
Z23	Mouse	IgG2b	+	+
Z24	Mouse	IgG2b	+	+
Z25	Mouse	IgG2b	+	+
Z26	Mouse	IgG2b	+	+
Z27	Mouse	IgG2b	+	+
2H12	Mouse	IgG3	+	+
12F2	Mouse	IgG3	+	+

Complete list of all human and murine anti-ZIKV NS1 mAbs tested.

[a] All anti-ZIKV NS1 human mAbs except for 749-A4 were derived from donor 973 in a previously published study [40]. Prior to blood donation and B cell collection, donor 973 had been infected with an African lineage strain of ZIKV (serum neutralization IC_{50} of 798) and immunized with the YFV vaccine but had no known DENV infection. Human mAb 749-A4 was isolated from a subject with a history of DENV infection and no other known flavivirus infections.

[b] Reactivity to recombinant, soluble ZIKV NS1 and murine IgG subclass were determined by ELISA. Human IgG subclass was determined by sequencing.

[c] Reactivity to ZIKV-infected C6/36 cells was determined by flow cytometry.

Supplementary Table 2. Characteristics of Anti-ZIKV NS1 mAbs

mAb[a]	Isotype[b]	Cross-reactivity[c]	EC_{50} (ng/ml) of Binding Cell Surface NS1[d]	Competition Group[e]	Binding to NS1 DII/III[f]	Domain Localization[g]	Critical Epitope Residues[g]
Z12	mIgG2a	none	29.2	A	No	Wing	R40, N82, W115
Z13	mIgG2a	none	105.7	A	No	Wing	R40, N82, W115
Z19	mIgG2a	none	66.2	A	No	Wing	N82
Z11	mIgG2a	none	2.2	B	No	Wing	Q102
Z15	mIgG2a	none	1.5	B	No	Wing	K146
Z18	mIgG2a	none	1.5	B	No	Wing	Q102
Z17	mIgG2a	none	18.4	C	Yes	β-platform	T290, R338
Z14	mIgG2a	none	1112.0	D	Yes	β-platform	G305, V307
Z16	mIgG2a	none	No binding	D	Yes		
Z20	mIgG2a	none	No binding	E	No	Wing	R40
ZIKV-292	hIgG1	none	3.4	B	No	Wing	P101, L177/E178
ZIKV-231	hIgG1	none	3.9	C	Yes	β-platform	K265, T293, R314
749-A4	hIgG1	W, J, D2	14.6	C	Yes	β-platform	E289, R314

[a] To generate murine mAbs, mice were inoculated and boosted with ZIKV (Dakar-MA) and then boosted with recombinant ZIKV NS1 protein. Human mAbs were isolated from subjects with a history of ZIKV (ZIKV-292 and ZIKV-231) or DENV (749-A4) infection.

[b] IgG subclass was determined by ELISA (m, murine; h, human).

[c] Cross-reactivity was determined by direct ELISA using recombinant NS1 proteins: W, WNV; J, JEV; D2, DENV2; Y, YFV; T, TBEV.

[d] EC_{50} value for binding to cell-surface NS1 was determined by flow cytometric analysis of ZIKV-infected cells, as described in **Figure 3**.

[e] Competition groups were determined by ELISA, as described in **Supplementary Table 3**.

[f] Binding to recombinant ZIKV NS1 DII/III protein (amino acids 172-352) was determined by ELISA.

[g] Domain localization was determined by binding to cells transfected with WT or mutant ZIKV NS1, as described in **Figure 4**. Critical epitope residues were defined as mutants with <25% binding to mAb compared to WT NS1.

Supplementary Table 3. MAb Competition Groups

Competition group		Second antibody													
		A			B				C			D		C/D	E
	mAb	Z12	Z13	Z19	Z11	Z15	Z18	ZIKV-292	Z17	ZIKV-231	749-A4	Z14	Z16	130.99	Z20
First antibody A	Z12	3	1	0	103	96	94	95	95	97	90	90	99	93	74
	Z13	14	5	3	92	95	87	92	94	96	99	89	100	93	74
B	Z15	86	82	71	4	2	0	13	90	93	100	88	92	82	70
	Z18	73	63	40	15	34	12	32	94	93	100	92	93	91	81
	ZIKV-292	112	60	71	4	5	1	1	86	96	85	93	93	97	114
C	Z17	53	35	43	87	89	86	85	1	30	2	94	85	1	53
	ZIKV-231	87	84	70	84	83	103	94	69	40	22	92	73	96	117
	749-A4	100	82	73	103	90	80	90	72	94	7	92	73	89	92
D	Z14	63	46	34	90	87	85	89	80	94	56	3	0	1	55

To determine mAb competition groups by ELISA, recombinant ZIKV NS1 protein was coated onto microtiter plates and incubated with the indicated first set of mAbs. Without washing, the indicated secondary set of mAbs (biotinylated) were added and after washing binding was detected using streptavidin-conjugated HRP. Values indicate percent of absorbance (OD450) signal detected relative to that obtained when the first antibody added was an irrelevant isotype control mAb.

Supplementary Table 4. Murine mAb Variable Region Sequencing

	Heavy chain						Kappa chain				
	Gene			CDR amino acid sequence			*Gene*		CDR amino acid sequence		
mAb	*V*	*D*	*J*	CDR1	CDR2	CDR3	*V*	*J*	CDR1	CDR2	CDR3
Z12	*IGHV5*	*IGHD2*	*IGHJ2*	GFTFS**S**YG	IS**G**GGIYT	**A**TYDYYFDF	*IGKV4*	*IGKJ2*	SSVSY	GIC	QQW**X**YRVIT
Z13	*IGHV5*	*IGHD2*	*IGHJ2*	GFTFS**T**YG	IS**T**GGIYT	**T**TYDYYFDF	*IGKV4*	*IGKJ2*	SSVSY	GIC	QQW**N**YRVIT
Z11	*IGHV1*	*IGHD3*	*IGHJ3*	GYTFTGYY	INPGTGYS	ARSGAHSGSIAY					
Z15	*IGHV1*	*IGHD3*	*IGHJ3*	GFTFTNSW	IHPGGGHV	ARTVWGFAF					
Z18	*IGHV1*	*IGHD1*	*IGHJ2*	GFTFTSSW	IHPNSGIT	ARLGYYGYVRDY					

The variable regions of the indicated murine antibodies were sequenced using previously described methods [77]. Total RNA was isolated from each hybridoma and cDNA was synthesized using SuperScript IV First Strain Synthesis kit. The heavy and light (kappa) chain variable regions were amplified using allele-specific primers and submitted for Sanger sequencing. For Z12 and Z13, differences in variable region amino acid sequences are indicated in red. One residue within the kappa chain CDR3 sequence for Z12 could not be resolved from the Sanger sequencing trace files and is indicated by a red X. For Z11, Z15, and Z18, the heavy chain variable region sequences were determined to be unique, so we did not proceed with sequencing of the light chain.

Supplementary Table 5. List of charge-reversal mutants

Mutant[a]	Z12[b]	Z13[b]	Z14[b]	Z15[b]	Z17[b]	Z18[b]	ZIKV-231[b]	ZIKV-292[b]	749-A4[b]	Oligo[c]
**W50R**	0.86	0.86	1.21	0.96	1.30	0.71	1.26	1.04	1.11	0.59
**E51R**	0.86	0.87	1.05	1.06	1.18	1.11	1.12	1.02	1.02	0.63
**D52R**	1.03	1.03	1.12	1.04	1.26	1.00	1.07	1.09	1.11	0.76
**ED51RR**	0.71	0.70	1.19	0.91	1.20	0.88	1.10	0.99	1.04	0.67
**E81R**	0.01	0.01	0.95	1.05	1.10	1.03	0.91	0.95	0.88	0.63
**G83R**	0.20	0.25	0.92	1.01	1.09	1.13	1.04	1.08	0.98	0.61
**Q85E**	0.96	0.93	0.90	1.10	1.14	1.14	1.06	1.06	0.95	0.61
**V89R**	0.93	0.87	0.85	0.99	0.99	1.09	0.85	0.93	0.94	0.66
**R99E**	1.05	1.06	1.01	0.96	1.00	0.94	1.02	0.91	0.99	1.05
**P101K**	0.97	1.01	0.91	0.85	0.87	0.69	0.78	0.01	0.81	1.04
**Q102R**	1.21	1.27	1.24	1.16	1.24	0.64	1.24	1.18	1.17	1.21
**Q102W**	1.06	1.05	1.08	0.96	1.24	0.03	1.14	1.06	1.04	0.72
**D138R**	0.76	0.65	0.98	1.00	1.05	1.21	1.12	1.00	0.96	0.53
**K146E**	1.23	1.27	1.26	0.00	1.24	1.27	1.25	1.12	1.24	1.17
**K146G**	1.05	0.84	1.06	0.65	1.20	0.97	0.98	0.98	0.91	0.65
**K170D**	0.92	0.90	1.09	1.06	1.13	1.07	1.10	1.01	1.07	0.82
**E173K**	1.22	1.23	1.23	1.05	1.12	1.23	1.23	1.01	1.12	1.18
**D174K**	1.20	1.17	1.22	1.04	1.20	1.07	1.13	0.97	1.18	1.15
**ED173KK**	1.04	0.96	1.10	0.99	1.10	1.01	1.04	0.71	0.98	0.92
**Y175K**	1.20	1.27	1.32	1.08	1.23	1.19	1.36	1.01	1.21	1.18
**L177K**	1.16	1.16	1.13	1.00	1.07	1.02	1.09	1.02	1.14	1.14
**E178M**	0.87	0.80	0.80	0.83	0.80	0.71	1.01	0.82	1.00	0.97
**E178K**	1.19	1.23	1.12	1.01	1.14	0.93	0.90	0.65	1.20	1.09
**E178R**	1.36	1.27	1.27	1.12	1.22	1.43	1.19	0.63	1.17	0.78
**LE177RR**	1.27	1.28	1.20	0.87	1.03	1.04	0.98	0.00	1.01	0.91
**K191D**	0.76	0.73	0.87	1.10	0.99	1.25	0.79	0.89	0.84	0.40
**D208K**	1.17	1.21	1.11	1.07	1.16	1.03	1.02	0.93	1.07	1.12
**T209K**	1.01	1.03	1.00	0.99	0.97	0.97	0.97	0.90	1.03	1.08
**T209E**	1.13	1.14	1.05	1.10	1.11	0.89	1.16	1.02	1.14	1.15
**DT208RR**	0.92	0.78	1.27	1.13	1.21	1.17	1.10	1.13	1.13	0.74
**K227S**	0.96	0.87	0.82	0.84	0.84	0.84	0.94	0.93	0.90	0.93
**K227E**	1.21	1.22	1.15	1.12	1.12	1.17	1.10	0.96	1.26	1.14
**W232D**	0.82	0.79	0.74	0.91	0.80	0.73	0.93	0.88	0.88	0.98
**G235K**	1.13	1.11	1.17	1.02	1.15	1.18	1.20	0.93	1.12	1.17
**E237R**	0.70	0.83	0.73	1.04	0.91	1.20	0.58	0.98	0.63	0.71
**E238K**	1.14	1.10	1.09	0.93	1.06	0.97	1.03	0.91	0.95	1.17
**K245E**	0.78	0.75	0.88	0.91	0.94	1.00	0.93	0.84	0.82	0.80
**L251K**	0.77	0.63	0.70	0.75	0.78	0.77	0.93	0.83	0.87	0.91
**E258D**	0.84	0.68	0.77	0.87	0.80	0.82	0.89	0.86	0.86	0.93
**E258K**	1.25	1.32	1.36	1.11	1.30	1.20	1.33	1.04	1.37	1.30
**E289K**	1.03	1.02	1.08	1.01	0.83	0.92	0.84	1.01	0.00	0.95
**T290K**	1.11	1.13	1.05	1.01	0.00	1.06	1.04	1.01	0.86	1.09
**G292D**	0.91	0.79	0.85	1.08	0.98	0.80	0.94	0.96	1.10	0.83
**T293K**	0.95	0.97	1.11	0.97	0.99	0.86	0.00	1.00	0.79	0.97
**A303R**	1.01	0.99	1.00	1.02	1.06	1.11	1.07	0.97	1.02	1.13
**S304R**	0.63	0.57	0.62	1.02	0.92	1.12	0.95	0.94	0.63	0.59
**G305K**	1.33	1.27	0.09	1.09	1.22	1.53	1.29	1.17	1.20	0.75
**R306D**	0.98	0.97	0.73	1.03	1.09	1.11	1.12	1.05	0.86	1.08
**V307R**	1.32	1.25	0.00	1.06	1.09	1.51	1.52	1.13	1.18	0.82
**E309R**	0.78	0.80	0.39	1.04	0.96	1.15	0.93	0.98	0.87	0.91

**E309W**	1.43	1.35	0.55	1.08	1.26	1.65	1.33	1.15	1.32	0.67
**E310R**	0.47	0.40	0.76	0.92	0.02	1.05	0.88	0.85	0.62	0.64
**E315K**	0.66	0.77	0.72	1.01	0.86	0.84	0.36	0.92	0.19	0.61
**K326D**	0.48	0.40	0.79	0.89	0.83	0.82	0.67	0.82	0.67	0.88

List of charge-reversal mutants used for epitope mapping.
[a] The indicated mutants were engineered in the pFM-A1.2 expression vector for ZIKV NS1 and transfected into 293T cells, and the mAb reactivity to each mutant relative to WT NS1 was measured by flow cytometry as described in **Figure 4**.
[b] For each mutant, the relative mAb reactivity was normalized to the staining of an oligoclonal mAb cocktail. Critical residues were defined as those mutants with <25% binding compared to WT NS1 (red, <25%; yellow, <50%).
[c] The oligoclonal antibody pool reactivity to each mutant relative to WT NS1 is shown. Mutants with <70% binding relative to WT (orange) of the oligoclonal antibody pool were considered poorly expressed and excluded from epitope mapping analysis.
