# Supplementary material for: Antibodies targeting epitopes on the cell-surface form of NS1 protect against Zika virus infection during pregnancy
Source: Nat Commun. 2020 Oct 19;11:5278. doi: 10.1038/s41467-020-19096-y (PMC7572419; doi:10.1038/s41467-020-19096-y)
Supplement: Supplementary file 2 — Description of Additional Supplementary Files [file 41467_2020_19096_MOESM2_ESM.pdf]

Description of Additional Supplementary Files

Supplementary Data 1. MAb reactivity to alanine-scanning library.

[a] The indicated mutants were engineered in the pFM-A1.2 expression vector for ZIKV NS1 and transfected into 293T cells, and the mAb reactivity to each mutant relative to WT NS1 was measured by flow cytometry as described in Figure 4. Cysteine residues are highlighted in grey.
[b] For each mutant, the relative mAb reactivity was normalized to the staining of an oligoclonal mAb cocktail. Critical residues were defined as those mutants with <25% binding compared to WT NS1 (red, <25%; yellow, <50%).
[c] The oligoclonal antibody pool reactivity to each mutant relative to WT NS1 is shown. Mutants with <70% binding relative to WT (orange) of the oligoclonal antibody pool were considered poorly expressed and excluded from epitope mapping analysis.

Supplementary Data 2. Antibody heavy- and light-chain gene sequences.

The nucleotide and corresponding amino acid sequences for *IGHV*, *IGKV*, and *IGLV* are shown for human mAb 749-A4.
