# Supplementary material for: Antibodies targeting epitopes on the cell-surface form of NS1 protect against Zika virus infection during pregnancy
Source: Nat Commun. 2020 Oct 19;11:5278. doi: 10.1038/s41467-020-19096-y (PMC7572419; doi:10.1038/s41467-020-19096-y)
Supplement: Supplementary file 5 — Reporting Summary [file 41467_2020_19096_MOESM5_ESM.pdf]

nature research

Corresponding author(s): Michael Diamond

Last updated by author(s): Sep 10, 2020

Reporting Summary

Nature Research wishes to improve the reproducibility of the work that we publish. This form provides structure for consistency and transparency in reporting. For further information on Nature Research policies, see Authors & Referees and the Editorial Policy Checklist .

Statistics

For all statistical analyses, confirm that the following items are present in the figure legend, table legend, main text, or Methods section.

n/a	Confirmed	
☐	☒	The exact sample size (*n*) for each experimental group/condition, given as a discrete number and unit of measurement
☐	☒	A statement on whether measurements were taken from distinct samples or whether the same sample was measured repeatedly
☐	☒	The statistical test(s) used AND whether they are one- or two-sided<br>*Only common tests should be described solely by name; describe more complex techniques in the Methods section.*
☒	☐	A description of all covariates tested
☐	☒	A description of any assumptions or corrections, such as tests of normality and adjustment for multiple comparisons
☐	☒	A full description of the statistical parameters including central tendency (e.g. means) or other basic estimates (e.g. regression coefficient) AND variation (e.g. standard deviation) or associated estimates of uncertainty (e.g. confidence intervals)
☐	☒	For null hypothesis testing, the test statistic (e.g. *F*, *t*, *r*) with confidence intervals, effect sizes, degrees of freedom and *P* value noted<br>*Give P values as exact values whenever suitable.*
☒	☐	For Bayesian analysis, information on the choice of priors and Markov chain Monte Carlo settings
☒	☐	For hierarchical and complex designs, identification of the appropriate level for tests and full reporting of outcomes
☒	☐	Estimates of effect sizes (e.g. Cohen's *d*, Pearson's *r*), indicating how they were calculated

Our web collection on statistics for biologists contains articles on many of the points above.

Software and code

Policy information about availability of computer code

Data collection	No software was used for data collection.
Data analysis	Statistical tests for each data set in this study were performed using GraphPad Prism (version 8.3) software. The crystal structures shown in Fig 4e-f were rendered using PyMOL (version 2.4) software. The sequence alignment in Fig 4g was rendered using ESPript (version 3.0) open-source software. FlowJo software was used for analysis of all flow cytometry data.

For manuscripts utilizing custom algorithms or software that are central to the research but not yet described in published literature, software must be made available to editors/reviewers. We strongly encourage code deposition in a community repository (e.g. GitHub). See the Nature Research guidelines for submitting code & software for further information.

Data

Policy information about availability of data

All manuscripts must include a data availability statement. This statement should provide the following information, where applicable:

- Accession codes, unique identifiers, or web links for publicly available datasets
- A list of figures that have associated raw data
- A description of any restrictions on data availability

The authors declare that all data supporting the findings of this study are available within the paper and its Supplementary information files, or from the corresponding author upon request. Source data underlying Figures 1f, 1h, 3b-g, and 4a-d and Supplementary Figures 1a-d and 3a-b are provided as a separate Source Data file.

Field-specific reporting

Please select the one below that is the best fit for your research. If you are not sure, read the appropriate sections before making your selection.

- [x] Life sciences
- [ ] Behavioural & social sciences
- [ ] Ecological, evolutionary & environmental sciences

For a reference copy of the document with all sections, see nature.com/documents/nr-reporting-summary-flat.pdf

Life sciences study design

All studies must disclose on these points even when the disclosure is negative.

Sample size	In the design of experiments, sample size calculations were included to distinguish 5-fold differences in viral burden
Data exclusions	None
Replication	For animal studies, each antibody treatment group was tested in at least two (Fig 1a-b, Fig 1e-h) or three (Fig 1c-d, Fig 2a-f) independent experiments, and always with an isotype control antibody treatment group to ensure consistency of viral titers across experiments. Antibody binding property, epitope mapping, and effector function data were also independently repeated in at least two (Fig 3d-g, Supplementary Fig 1a-d, Supplementary Fig 3a-b) or three (Fig 3a-c, Fig 4a-d) experiments and also included appropriate isotype controls to ensure reproducibility. Attempts at replication were successful.
Randomization	For animal studies, mice were randomly assigned to antibody treatment groups. For studies in non-pregnant hSTAT2-KI mice, we attempted to use equivalent numbers of male and female mice for each treatment group. For in vitro antibody studies, randomization was not relevant since the same original pool of cells was used within an experiment for all virus infections, NS1-mutant transfections, and effector function studies.
Blinding	Blinding was not relevant to our study because none of the data was acquired by manual human measurement. Viral titer data were acquired using an RT-qPCR instrument. Antibody binding property, epitope mapping, and effector function data were acquired using a flow cytometer or ELISA plate reader.

Reporting for specific materials, systems and methods

We require information from authors about some types of materials, experimental systems and methods used in many studies. Here, indicate whether each material, system or method listed is relevant to your study. If you are not sure if a list item applies to your research, read the appropriate section before selecting a response.

Materials & experimental systems

n/a	Involved in the study	
	x	Antibodies
	x	Eukaryotic cell lines
x		Palaeontology
	x	Animals and other organisms
	x	Human research participants
x		Clinical data

Methods

n/a	Involved in the study	
x		ChIP-seq
	x	Flow cytometry
x		MRI-based neuroimaging

Antibodies

Antibodies used	All new mouse and human anti-NS1 mAbs generated in this study are listed in Tables S1 and S2. Other antibodies used: anti-CD3 (BD Pharmingen #345766); anti-CD19 (Dako #R0808); anti-CD20 (BD Pharmingen #345794); anti-CD27 (BD Pharmingen #555440); anti-CD38 (BD Pharmingen #555462); anti-Ifnar1 blocking mAb (Leinco clone MAR1-5A3 #I-401); anti-CD11b APC (BioLegend clone M1/70 #101212); CD11c APC/Cy7 (BioLegend clone N418 #117324); Ly6G Pacific Blue (BioLegend clone 1A8 #127612); Ly6C BV605 (BioLegend clone HK1.4 #128036); CD3 PE/Cy7 (BioLegend clone 17A2 #100220); anti-guinea pig C3b FITC (MP Biomedicals 0855385)
Validation	Each anti-NS1 mAb was validated for binding to recombinant, purified NS1 by ELISA or BLI and staining of NS1-transfected cells by flow cytometry. The commercial antibodies were validated by their respective manufacturers.

Eukaryotic cell lines

Policy information about cell lines

Cell line source(s)	293T, Vero, C6/36, P3X63 Ag8.653, J774A.1, and B95-8 cells were from ATCC. Hybridomas were generated in the Diamond laboratory.

Authentication	These were obtained from ATCC and grew and performed as expected; however, they were not independently authenticated in our laboratory.
Mycoplasma contamination	All lines are routinely screened and have tested negative for mycoplasma using a Core facility at Washington University.
Commonly misidentified lines (See ICLAC register)	This study did not involve any commonly misidentified cell lines.

Animals and other organisms

Policy information about studies involving animals; ARRIVE guidelines recommended for reporting animal research

Laboratory animals	Human STAT2-knock in mice (C57BL/6J background): males, 3-4 weeks; females, 3-4 weeks and 8-16 weeks. BALB/c mice: females, 6 weeks.
Wild animals	No wild animals were used in this study.
Field-collected samples	No field-collected samples were used in this study.
Ethics oversight	All animal procedures were carried out in accordance with the recommendations in the Guide for the Care and Use of Laboratory Animals of the National Institutes of Health. The protocols were approved by the Institutional Animal Care and Use Committee at the Washington University School of Medicine (assurance no. A3381-01).

Note that full information on the approval of the study protocol must also be provided in the manuscript.

Human research participants

Policy information about studies involving human research participants

Population characteristics	The human participant was identified as Zika virus immune by history of infection and a screening test for pre-existing antibodies. For the Dengue studies, blood samples were collected from inpatients after written informed consent. Laboratory confirmation of DENV infection was determined by RT-PCR detection of DENV nucleic acid (which also confirmed the infecting serotype).
Recruitment	The Zika-immune individual who provided PBMCs for these studies was an otherwise healthy adult female in the U.S. who was infected with an African lineage strain in 2008 by sexual transmission from a contact who was infected in Senegal, as previously reported:<br><br>Foy, B. D. et al. Probable non-vector-borne transmission of Zika virus, Colorado,<br>USA. Emerg. Infect. Dis. 17, 880–882 (2011).<br>And<br>Sapparapu G, Fernandez E, Kose N, Cao B, Fox JM, Bombardi RG, Zhao H, Nelson CA, Bryan AL, Trevor Barnes, Davidson E, Mysorekar IU, Fremont DH, Doran BJ, Diamond MS, Crowe JE Jr. Neutralizing human antibodies prevent Zika virus replication and fetal disease in mice. Nature 2016; 540: 443 – 447. PMC ID: PMC5583716.
Ethics oversight	The Vanderbilt University Medical Center Institutional Review Board approved the protocol for obtaining blood samples from immune individuals for generating human monoclonal antibodies. Samples were obtained only after written informed consent. The Dengue study protocol was approved by the Scientific and Ethical Committee of the Hospital for Tropical Diseases, the Oxford Tropical Research Ethical Committee and the Riverside Ethics Committee in the United Kingdom.

Note that full information on the approval of the study protocol must also be provided in the manuscript.

Flow Cytometry

Plots

Confirm that:

- [x] The axis labels state the marker and fluorochrome used (e.g. CD4-FITC).
- [x] The axis scales are clearly visible. Include numbers along axes only for bottom left plot of group (a 'group' is an analysis of identical markers).
- [x] All plots are contour plots with outliers or pseudocolor plots.
- [x] A numerical value for number of cells or percentage (with statistics) is provided.

Methodology

Sample preparation	Supernatants were screened for antibody binding to ZIKV-infected cells by flow cytometry or to recombinant ZIKV NS1 protein by ELISA. Briefly, ZIKV-infected C6/36 cells (MOI of 0.1, 3 dpi) were fixed with 4% paraformaldehyde (PFA), permeabilized in PBS, 0.1% saponin, 0.1% bovine serum albumin (BSA), and then incubated with hybridoma culture supernatants supplemented with 0.1% saponin. Anti-NS1 mAbs were detected using Alexa Fluor 647-conjugated goat anti-mouse IgG (1:2000 dilution; Thermo

	Fisher).
Instrument	Miltenyi Biotec MACSQuant Analyzer 10; IntelliCyt iQue Screener Plus; 3L Stratedigm S1300EXI
Software	FlowJo
Cell population abundance	No sorting was performed
Gating strategy	Live cells were distinguished via a live/dead gate. Singlets were determined using FSC/SSC.

☒ Tick this box to confirm that a figure exemplifying the gating strategy is provided in the Supplementary Information.
